# Uncovering receptor-ligand interactions using a high-avidity CRISPR activation screening platform

**DOI:** 10.1126/sciadv.adj2445

**Published:** 2024-02-14

**Authors:** Liping Yang, Timothy P. Sheets, Yang Feng, Guojun Yu, Pradip Bajgain, Kuo-Sheng Hsu, Daeho So, Steven Seaman, Jaewon Lee, Ling Lin, Christine N. Evans, Mary R. Guest, Raj Chari, Brad St. Croix

**Affiliations:** ^1^ Tumor Angiogenesis Unit, Mouse Cancer Genetics Program (MCGP), National Cancer Institute (NCI), NIH, Frederick, MD 21702, USA.; ^2^ Genome Modification Core, Laboratory Animal Sciences Program, Frederick National Lab for Cancer Research, Frederick, MD 21702, USA.; ^3^ Proteomic Instability of Cancer Section, MCGP, NCI, NIH, Frederick, MD 21702, USA.

## Abstract

The majority of clinically approved drugs target proteins that are secreted or cell surface bound. However, further advances in this area have been hindered by the challenging nature of receptor deorphanization, as there are still many secreted and cell-bound proteins with unknown binding partners. Here, we developed an advanced screening platform that combines CRISPR-CAS9 guide–mediated gene activation (CRISPRa) and high-avidity bead-based selection. The CRISPRa platform incorporates serial enrichment and flow cytometry–based monitoring, resulting in substantially improved screening sensitivity for well-known yet weak interactions of the checkpoint inhibitor family. Our approach has successfully revealed that siglec-4 exerts regulatory control over T cell activation through a low affinity trans-interaction with the costimulatory receptor 4-1BB. Our highly efficient screening platform holds great promise for identifying extracellular interactions of uncharacterized receptor-ligand partners, which is essential to develop next-generation therapeutics, including additional immune checkpoint inhibitors.

## Teaser

Combining CRISPRa with magnetic bead-based enrichment unveils an improved method for receptor deorphanization.

## INTRODUCTION

Cell surface protein-protein interactions are crucial for various cellular functions, including cell growth, cell-cell communication, signal transduction, and immune responses (*1*) and are also an essential target node for controlling disease-related signaling networks (*2*, *3*). Approximately 70% of US Food and Drug Administration–approved therapeutic agents target proteins that are either secreted or cell surface bound (*4*, *5*). For example, immune checkpoint inhibitors such as antibodies targeting cytotoxic T lymphocyte–associated antigen 4 (CTLA4), programmed cell death-1 (PD-1), and programmed cell death ligand 1 (PD-L1) are established agents used to reinvigorate antitumor immunity (*6*). Despite their proven utility as drug targets, many physiologic extracellular ligand-receptor interactions remain uncharacterized.

Receptor deorphanization poses a major challenge primarily due to the prevalence of low-affinity interactions in the micromolar range and rapid dissociation rates among many extracellular ligand-receptor pairs (*7*–*9*). Although these weak interactions may be favored biochemically because they enable rapid, transient, and reversible responses to stimuli at the cell surface (*9*–*11*), they pose a challenge for creating high-throughput screens to identify new ligand-receptor interactions. Another challenge stems from the hydrophobic transmembrane domains present in receptors, which make it difficult to solubilize full-length proteins in a native conformation.

One successful method used to deorphanize receptors involves multimerizing individual ectodomain (ED) “prey” proteins on a solid surface and probing them with candidate “bait” proteins (*1*, *12*). This approach enhances the avidity of transient interactions. However, scaling up for high-throughput screening is complicated due to the extensive resources required for mass protein production and purification, making this strategy challenging for many laboratories. This approach is also unsuitable for G protein–coupled receptors and other multipass membrane proteins with discontinuous ligand binding sites spanning multiple EDs. While cDNA expression libraries encoding thousands of cell surface proteins provide a cell-based system that can address this problem, such screens are also resource intensive, requiring thousands of plasmid vectors and limiting accessibility for most laboratories (*13*, *14*).

Recent advances in CRISPR-CAS9 guide–mediated gene activation (CRISPRa) provide a more accessible cell-based approach for receptor deorphanization, only requiring access to a single library of short guide RNAs (gRNAs) (*8*, *15*). Capitalizing on this approach, pioneering work by the Wright laboratory initially demonstrated that this method can be used to identify receptor-ligand interactions known or predicted to reside at the cell surface (*15*). By creating a library of barcoded cells, each overexpressing on average a single-cell surface receptor activated by one individual gRNA, recombinant ED bait proteins were then used to label and purify cells, which were then subjected to high-throughput sequencing. Intrigued by this approach, we tested the system using as bait the ED of PD-L1, a transmembrane spanning ligand known to bind the PD-1 surface receptor with an equilibrium dissociation constant (*K*_D_) of 7.2 μM (*16*). However, our initial attempt to uncover this interaction was unsuccessful, and from published literature, we noticed that three of the four known interactions validated in the original study have substantially higher affinities in the nanomolar range (*17*–*19*). To determine whether assay sensitivity could be improved, we created multimerized ED-conjugated bead baits, reasoning that the presentation of thousands of proximally oriented EDs would allow superior “Velcro-like” avidity in a configuration akin to natural receptor presentation on the cell surface. We also evaluated the impact of serial magnetic bead–based selection on enrichment kinetics, and provide a strategy to ascertain success before deep sequencing. Using this highly sensitive platform, we identify and characterize siglec-4 as a low-affinity binding partner for the costimulatory receptor 4-1BB (CD137/TNFRSF9) that can modulate 4-1BB immunoregulatory function on activated T cells, demonstrating the potential of the approach to help identify remaining uncharacterized low-affinity receptor-ligand interactions.

## RESULTS

### Evaluating the ability of CRISPRa to identify a known interacting partner

**Fig. 1.**
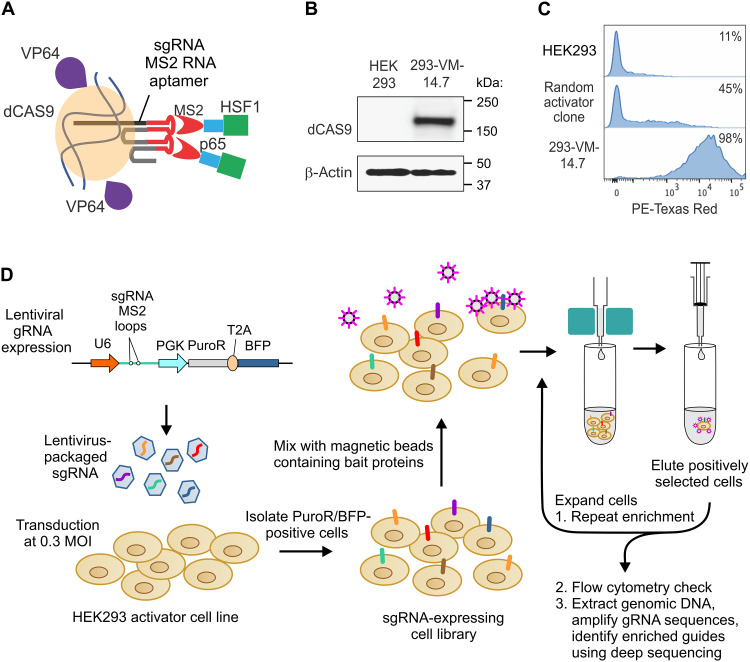
Schematic representation of avidity-based CRISPRa screen. (**A**) Depiction of the assembled dCAS9 transcriptional activator gRNA complex. (**B**) Western blot showing dCAS9 expression in the 293-VM-14.7 activator cells. (**C**) FC showing tdTomato expression in parental HEK293 cells and two activator clones following transfection with gRNA tdTomato reporter. (**D**) Schematic representation of the steps used for high–avidity-based CRISPRa screening.

CRISPRa was recently introduced as a new strategy to deorphanize cell surface receptors (*15*). To test this system, we created a human embryonic kidney (HEK) 293 activator cell line by stably introducing endonuclease dead CAS9 (dCAS9) fused with the transcriptional activator VP64 (Fig. 1A). Gene-specific transcriptional activation in this system is facilitated by the coexpression of an MS2-p65-HSF1 fusion protein, where the MS2 subunit binds to the RNA hairpin loops adjacent to individual gRNAs (*20*). Upon cloning by single-cell dilution, a dCas9^+^ HEK293 activator clone (293-VM-14.7) capable of inducing the tdTomato reporter (*21*) in the majority of cells was identified (Fig. 1, B and C).

The original gRNA library from the Wright laboratory contains 58,071 guides designed to target promoter regions of 6213 genes encoding proteins with transmembrane-spanning regions or secreted proteins associated with the plasma membrane (*15*). The library was packaged into viral particles and transduced into 293-VM-14.7 cells at a multiplicity of infection (MOI) of 0.3, helping ensure that following puromycin selection most cells overexpressed a single gene. Deep sequencing verified that the gRNA library maintained over 95% of its diversity following amplification in *Escherichia coli* and subsequent transduction into 293-VM-14.7 cells (fig. S1 and tables S1 and S2).

To test the system, we used the ED of PD-L1 as bait, as the PD-L1–PD-1 ligand-receptor interaction has been well established and extensively targeted in the clinic using immune checkpoint inhibitors (*22*, *23*). Guided by the original method, the PD-L1–ED bait was biotinylated, labeled with Alexa Fluor 488 (AF488)–streptavidin and used to isolate the top 5% of cells using fluorescence-activated cell sorting (FACS). Genomic DNA was then isolated from sorted cells and subjected to deep sequencing. However, we failed to identify any *PDCD1* (PD-1) gRNAs from the top sorted cells. Using flow cytometry (FC), we verified that most of the individual *PDCD1 *guides present in the library could induce PD-1 expression in activator cells, although expression levels varied depending on the particular guide (fig. S2A). Notably, while the original study detected an interaction between CTLA4 and CD80 (*K*_D_: 200 nM), it did not provide evidence for the interaction between CTLA4 and its lower affinity binding partner CD86 (*K*_D_: 2.6 μM) (*15*, *24*), an affinity closer to that of PD-1/PD-L1 (*K*_D_: 7.2 μM) (*16*). This was also unlikely due to ineffective guides, as we verified the successful expression of CD86 after the transduction of *CD86*-specific gRNAs (fig. S2B). To facilitate the detection of well-established but relatively low-affinity interactions in the micromolar range, we decided to explore different approaches to increase assay sensitivity.

### Optimization of the screening platform

Biochemical screening platforms using purified recombinant proteins as probes have revealed that high avidities of both bait and prey proteins are crucial for optimal sensitivity (*12*). However, the streptavidin-biotin-bait proteins used in the previous Wright study contain only four copies of biotin-bait per streptavidin complex (*15*). Furthermore, the opposing orientation of biotin binding sites on streptavidin potentially imposes physical constrains on the bound bait proteins, limiting their simultaneous binding to the target (*25*, *26*). To address this issue, we armed nano-sized magnetic beads with the ED of bait proteins, presenting thousands of baits in close proximity and enabling the detection of low-affinity interactions. To assess the versatility and efficiency of this system, we compared protein A–, anti–HIS–monoclonal antibody (mAb)–, and streptavidin-linked magnetic beads bound to CTLA4 or PD-L1 bait proteins fused to Fc, 6xHIS, or biotin binding peptide tags at the C terminus of their EDs.

**Fig. 2.**
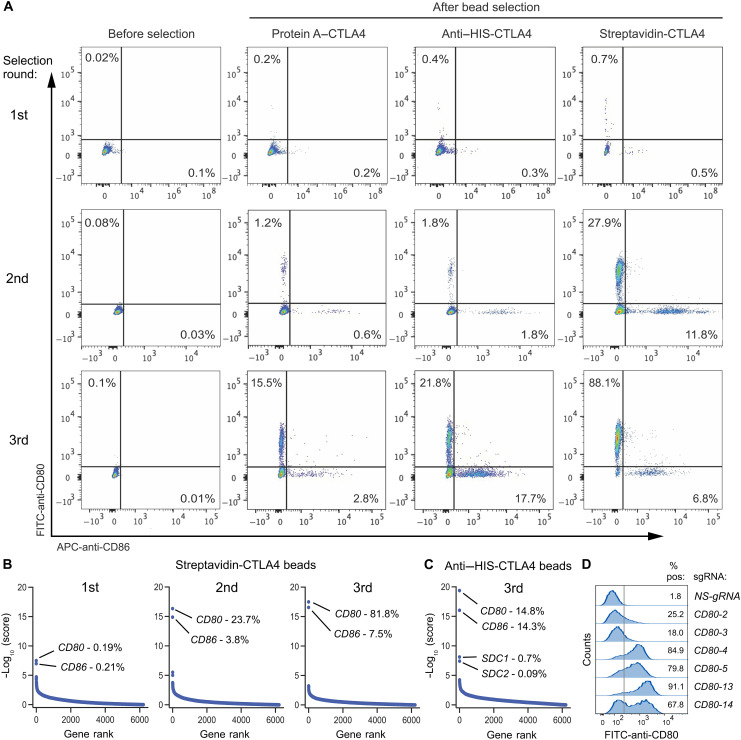
Serial enrichment of CD80 and CD86 using CTLA4 beads. (**A**) FC was used to monitor CD80 and CD86 enrichment in activator cells following three rounds of selection using protein A–, anti-HIS-, and streptavidin-CTLA4 magnetic beads. Data were representative of three experiments. (**B**) The gRNA enrichment following first, second, or third round of selection using streptavidin-CTLA4 magnetic beads was monitored using −log_10_ (score). (**C**) The gRNA enrichment following the third round of selection using anti-HIS-CTLA4 magnetic beads was monitored using −log_10_ (score). The percentages in (B) and (C) indicate, for each library, the total gRNA counts for the gene of interest divided by the total number of gRNA counts for all genes (×100). (**D**) FC was used to monitor surface expression of CD80 48 hours following transduction with individual CD80 gRNAs amplified during enrichment. Data were representative of three experiments.

To ensure a sufficient number of cells (>1 million) for the subsequent gRNA isolation and analysis, the prior Wright protocol isolated the top 5% of streptavidin-bait–labeled cells by FACS. However, there was no clear evidence of increased bait binding over nontransduced control cells alone (*15*), suggesting limited detectability of target enrichment by FC after a single round of selection. Therefore, we assume that enrichment from this original method is only detectable in the sorted cells at the gRNA level following deep sequencing. To further enhance assay sensitivity, we subsequently performed up to five serial enrichments using magnetic-activated cell sorting (MACS), expanding the sorted cells between each selection. Our modified technique, outlined in Fig. 1D, involved expanding and dividing all bead-bound cells after each round of MACS for genomic DNA (gDNA) isolation, FC monitoring, or further MACS. Less rinsing was applied in the initial two rounds of MACS to minimize rare gRNAs loss before amplification. Gratifyingly, using CLTA4 bait protein for serial MACS, we successfully enriched both known binding partners, CD80 and CD86, as verified by FC detection with monoclonal antibodies (Fig. 2A). Furthermore, the comparison among protein A–, anti–HIS-mAb–, and streptavidin-linked magnetic beads revealed that all three CTLA4 bait formats effectively enriched both targets, with streptavidin-linked magnetic beads showing the highest efficiency. Consistent with the FC analysis, deep sequencing of the streptavidin and anti-HIS libraries confirmed the *CD80* and *CD86* enrichment, as evident when gene-level enrichment scores were plotted against gene rank order (Fig. 2, B and C). FC analysis confirmed that the individual *CD80* (Fig. 2D) and *CD86* (fig. S2B) gRNAs enriched in the libraries were capable of inducing expression of CD80 and CD86 in 293-VM-14.7 activator cells. These results reveal improved sensitivity through bead-based selection.

**Fig. 3.**
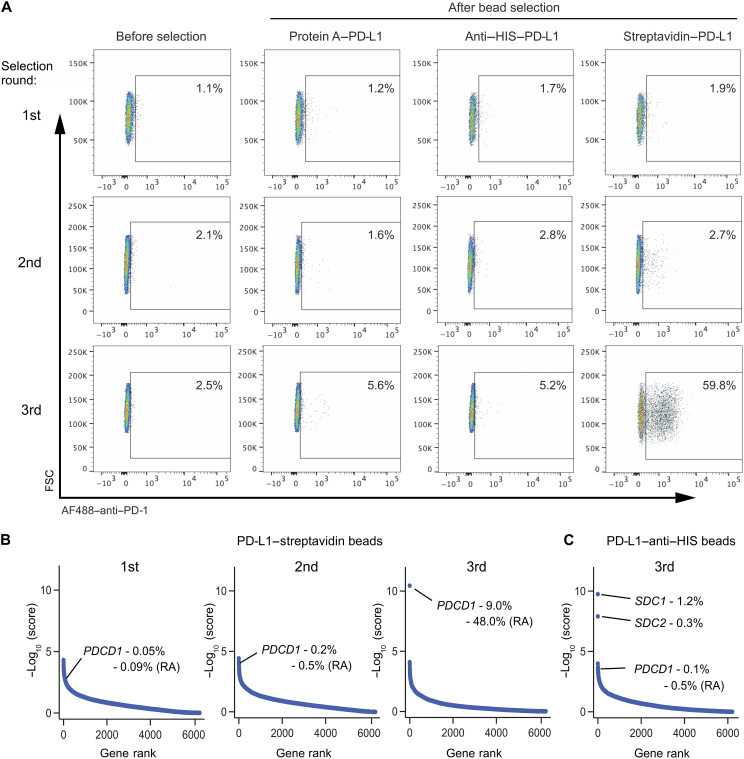
PD-L1 beads facilitate the serial enrichment of PD-1. (**A**) FC was used to monitor PD-1 enrichment in activator cells following three rounds of selection using protein A–, anti-HIS–, and streptavidin–PD-L1 magnetic beads. Data were representative of three experiments. (**B**) The gRNA enrichment following first, second, or third round of selection using streptavidin–PD-L1 magnetic beads was monitored using −log_10_ (score). (**C**) The gRNA enrichment following the third round of selection using anti–HIS–PD-L1 magnetic beads was monitored using −log_10_ (score). The percentages in (B) and (C) indicate, for each library, the total gRNA counts for the gene of interest divided by the total number of gRNA counts for all genes (×100). RA, re-annotated *PDCD1* gRNA frequencies following reassignment of *WNT7A-6* and *ICA1-4* to *PDCD1* gRNAs.

Next, we evaluated the ability of PD-L1 to identify its established binding partner PD-1 using protein A–, anti–HIS-mAb-, and streptavidin–PD-L1–linked magnetic beads. As shown in Fig. 3A, PD-1 enrichment could be readily identified by FC using all three formats of PD-L1 beads. As with the CTLA4 bait, the PD-L1–streptavidin beads were the most efficient at enriching PD-1, which was expressed in approximately 60% of the total cell population by the third round of enrichment (Fig. 3A). Deep sequencing of each round of the PD-L1–streptavidin libraries revealed that *PDCD1* emerged as the top hit after the third enrichment when transformed gene-level enrichment scores were plotted against gene rank order (Fig. 3B). In this library enrichment, *PDCD1* guides constituted 0.2% of total guide counts by the second sort and 9% of guides by the third sort. Deep sequencing of the PD-L1–HIS libraries confirmed the inferior effectiveness of these magnetic beads compared to streptavidin beads, consistent with the FC data (Fig. 3, A and C).

While antibodies against CD80, CD86, and PD-1 provide a convenient tool to assess target enrichment following serial sorting with CTLA4- or PD-L1–linked beads, such antibodies would not be available when searching for an unknown binding partner. Therefore, magnetic beads dual labeled with fluorescein isothiocyanate (FITC) and bait protein (i.e., CTLA4-ED or PD-1–ED) were used to stain the corresponding cells following each round of sorting, to monitor enrichment. Notably, dual-labeled beads exhibited clear binding, similar to that observed with antibodies against the known targets (compare fig. S3, A and B, with the third selection from Figs. 2A and 3A). This indicates that the bait proteins themselves can serve as a reliable indicator of successful enrichment. Furthermore, the level of enrichment can guide the decision to halt serial MACS and determine whether sequencing only a few hundred guides would be sufficient for target identification. As deep sequencing represents the costliest part of this avidity-based deorphanization protocol, performing multiple rounds of enrichment can not only enhance the screening efficiency but also substantially reduce overall expenses.

### Generation of a more stringent gRNA library

**Fig. 4.**
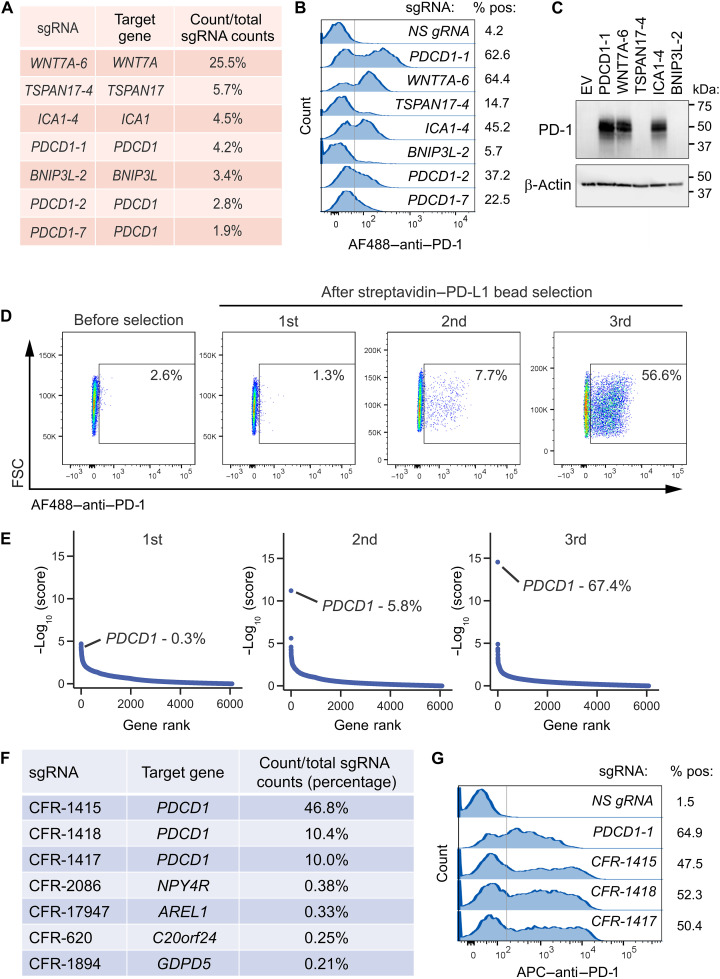
Library gRNA annotation and *PDCD1* guide enrichment using stringent guide design. (**A**) Table showing the top seven individual gRNAs in decreasing order of abundance following the third selection of the original library with streptavidin–PD-L1 magnetic beads. (**B**) FC was used to assess which of the top guides can turn on PD-1 expression. Data were representative of three experiments. (**C**) Western blotting of PD-1 in 293-VM-14.7 activator cells engineered to express the top gRNAs. Data were representative of three experiments. (**D**) FC was used to monitor PD-1 enrichment in activator cells containing the new stringent CRISPRa library following up to three rounds of selection with streptavidin–PD-L1 magnetic beads. Data were representative of three experiments. (**E**) The gRNA enrichment of the new library following three rounds of selection using streptavidin–PD-L1 magnetic beads was monitored using −log_10_ (score). (**F**) Table showing the top seven individual gRNAs in decreasing order of abundance following the third selection of the new stringent library with streptavidin–PD-L1 magnetic beads. The percentages in (A), (E), and (F) indicate, for each library, the total gRNA counts for the gene of interest divided by the total number of gRNA counts for all genes (×100). (**G**) FC was used to verify that the three top *PDCD1* guides enriched in the new CheriFNL-Relaxed (CFR) library can turn on PD-1 expression. Data were representative of three experiments.

While FC analysis and deep sequencing generally showed consistent results in terms of target and gRNA enrichment, some gRNA enrichment rates were lower than expected. For example, following three rounds of enrichment using streptavidin–PD-L1 beads, FC revealed ~60% PD-1–positive cells, while the deep sequencing revealed that *PDCD1* gRNAs only accounted for 9% of total guides (compare streptavidin bead selections from Fig. 3, A and B). Unexpectedly, the top gRNA enriched by the PD-L1 bait–coated beads was a *WNT7A* gRNA (*WNT7A-6*) rather than *PDCD1* gRNA and accounted for 25.5% of all the guides by the third round of selection (Fig. 4A). Closer inspection revealed that 13 of 20 contiguous nucleotides of *WNT7A-6* gRNA shared 100% identity with a region in the *PDCD1* promoter 585 nucleotides upstream of the ATG start codon, suggesting that 13 nucleotides may be sufficient to bind and activate the *PDCD1* promoter (*27*). Another highly enriched gRNA (*ICA1-4*), targeting *ICA1*, shared 16 of 20 contiguous nucleotides with the *PDCD1* promoter 467 bp upstream of the ATG start codon. To determine whether *WNT7A-6* and *ICA1-4* gRNAs could induce PD-1, lentiviral particles containing these gRNAs were used to transduce 293-VM-14.7 cells. We also tested gRNA at the top of the list (Fig. 4A) without a high level of homology to the *PDCD1* promoter. Similar to *PDCD1* gRNAs, the *WNT7A-6* and *ICA1-4* gRNAs could also induce robust PD-1 expression as detected by both FC and Western blotting (Fig. 4, B and C).

Another unexpected discovery we made while working with these libraries is that certain gRNAs were enriched with multiple different baits. For example, syndecan-1 (*SDC1*) and syndecan-2 (*SDC2*) guides appeared near the top of our gene lists for CTLA-4 and PDL1 anti-HIS beads (Figs. 2C and 3C). These guides also appeared in the original Wright analysis, where syndecans were implicated as putative binding partners for ADGRA2 (*15*), but without further validation. However, we failed to validate the interaction between syndecans and CTLA-4 using FC (fig. S4), suggesting that their enrichment may result from transient, ionic interactions. Syndecans are negatively charged heparan sulfate proteoglycans known to bind many other charged molecules (*28*, *29*). While syndecan gRNAs were enriched on baits appended to both anti-HIS and protein A beads, their levels remained near background on the streptavidin baited beads.

To improve accuracy in target identification, we opted to create a new gRNA library with increased stringency. Using a custom Python script and cross-referencing with GuideScan off-target monitoring (*30*), we identified and combined up to five highly specific gRNAs per gene from the Wright and the widely used Calabrese and Weissman libraries (see Materials and Methods for more details) (*15*, *31*, *32*). The resulting stringent library comprised 30,868 gRNAs targeting 6081 genes with 500 nontargeting controls (table S3). On average, each gene in the new library was represented by approximately 4.5 gRNAs compared to 9.3 in the Wright library (*15*).

After lentiviral transduction of the new library into 293-VM-14.7 cells and selection using puromycin, we tested the new library using streptavidin–PD-L1 magnetic beads as before. Following serial enrichment, four of the five *PDCD1* guides in the new library amplified and accounted for 67.4% of guides by the third sort, mirroring the FC enrichment data (Fig. 4, D to G). In contrast, only three of seven correctly assigned *PDCD1* gRNAs successfully amplified from Wright’s original library, representing 9.0% of total guides following the third enrichment (Fig. 3B). While manual re-annotation of the misassigned guides in the Wright library raised the percent positive *PDCD1* gRNAs to 48% (Fig. 3B), this process required additional wet lab validation (Fig. 4, B and C), making in silico correction challenging. In addition, the *PDCD1* gRNAs rose to the top after the second round of sorting using the new library (Fig. 4E), whereas three rounds were required using the Wright library (Fig. 3B). These data indicate that the binding partner of PD-L1 was enriched more efficiently in the new gRNA library.

### Identification of Siglec-4 as a 4-1BB binding partner

**Fig. 5.**
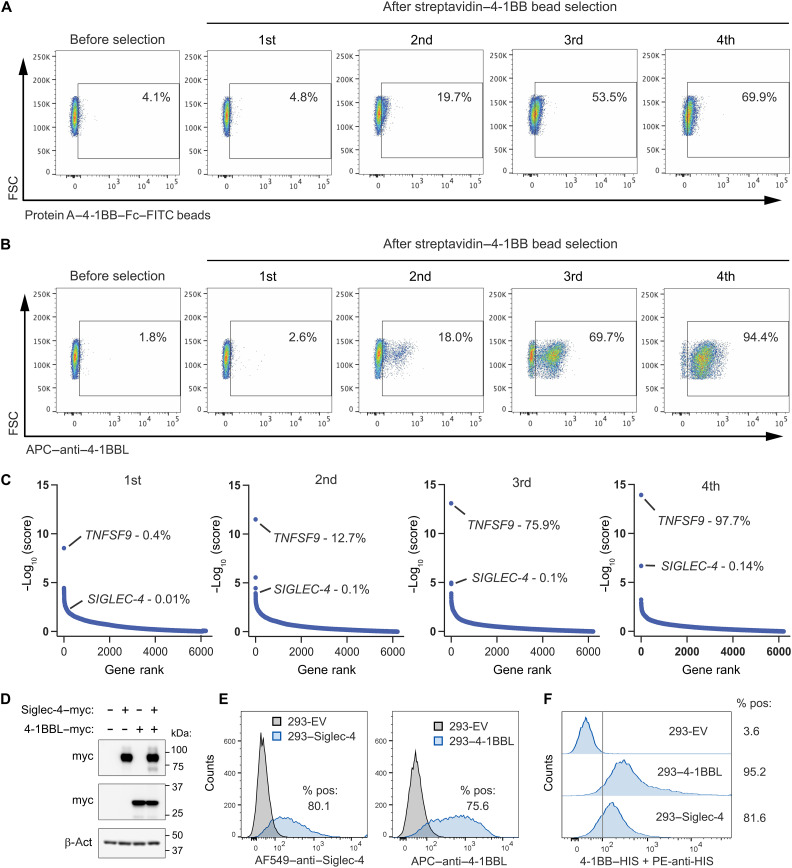
4-1BB beads facilitate the serial enrichment of 4-1BBL and siglec-4. (**A**) FC was used to monitor binding of FITC-labeled 4-1BB magnetic beads to activator cells following up to four rounds of selection using streptavidin–4-1BB magnetic beads. (**B**) FC was used to monitor 4-1BBL expression in activator cells following up to four rounds of enrichment with streptavidin–4-1BB magnetic beads. (**C**) The gRNA enrichment following first, second, third, or fourth round of selection using streptavidin–4-1BB magnetic beads was monitored using −log_10_ (score). For each library, the percentages indicate the total gRNA counts for the gene of interest divided by the total number of gRNA counts for all genes (×100). (**D**) Immunoblotting was used to detect myc in 293 cells transfected with siglec-4–myc or 4-1BB–myc. β-Actin (β-act) was included as a loading control. (**E**) FC was used to measure the level of siglec-4 or 4-1BBL on the surface of 293 cells following transfection with pCMV EV (control), pCMV–siglec-4 (siglec-4), or pCMV–4-1BBL (4-1BBL). (**F**) Binding of 4-1BB–HIS to 293 cells transfected with EV (negative control), pCMV–4-1BB (positive control), or pCMV–siglec-4. Data in (A), (B), (D), (E), and (F) were representative of three experiments.

4-1BB has long been recognized for its ability to promote immune stimulation and antitumor responses by binding strongly to the transmembrane 4-1BB ligand (4-1BBL/TNFSF9) (*K*_D_ = 85.6 nM) (*33*, *34*). However, 4-1BB agonistic antibodies with tumoricidal activity also unexpectedly improved outcomes in mouse models of autoimmune diseases (*35*–*38*), suggesting a more complex regulation and the possibility of additional binding partners. To explore the potential of our assay to identify previously uncharacterized 4-1BB binding partners, we used streptavidin–4-1BB–ED–coated beads for library selection. As expected, we observed increased cell binding with each round of sorting (Fig. 5A) and verified a strong enrichment of 4-1BBL by both FC monitoring and gRNA enrichment analysis (Fig. 5, B and C). *SIGLEC-4* (*MAG*) also steadily increased during sequential sorting and became, after *TNFSF9* (*4-1BBL*), the second highest hit by the fourth round of selection (Fig. 5C). Assessment of individual enriched guides revealed that *SIGLEC-4* gRNAs could induce cell surface siglec-4 protein but not 4-1BBL, verifying correct target specificity (fig. S5). To validate the 4-1BB/siglec-4 interaction, 4-1BB–HIS protein was used in FC to stain HEK293 cells overexpressing siglec-4, revealing strong binding compared to cells transfected with empty vector (EV) alone (Fig. 5, D to F). Their biophysical interaction was further verified using enzyme-linked immunosorbent assay (ELISA) and surface plasmon resonance (SPR) analysis (fig. S6, A to D). Despite the low conservation of 4-1BB among species, with mouse 4-1BB (m-41BB) and human 4-1BB (h-41BB) sharing only 61% amino acid identity in their EDs, SPR analysis revealed that m-41BB–ED bound to mouse siglec-4 (m-siglec-4) with an affinity similar to the human pair (m-41BB and m-siglec-4, *K*_D_: 0.86 μM; h-41BB and h-siglec-4, *K*_D_: 0.33 μM) (fig. S6, B to D). FC also verified the m-41BB/m-siglec-4 interaction (fig. S6, E and F). Coimmunoprecipitation studies further revealed a strong interaction between the receptors even when expressed in separate cells, corroborating the MACS data and indicating their in trans interaction (fig. S6G). Siglec-4 is a single-pass transmembrane protein of the siglec family of sialic acid binding proteins, which are broadly known for their immunomodulatory roles (*39*) and ability to interact with specific N-linked glycoproteins containing terminal *N*-acetyl-neuraminic acid (Neu5AC) (*40*). 4-1BB contains two N-linked carbohydrate side chains with terminal Neu5AC that do not affect its ability to bind 4-1BBL (*41*). We evaluated siglec-4 binding after 4-1BB deglycosylation but found no decrease in binding, suggesting that the interaction could occur without carbohydrate (fig. S7, A and B). Furthermore, competition studies revealed that siglec-4 could bind 4-1BB even in the presence of a 10-fold excess of 4-1BBL, indicating that the siglec-4 and 4-1BBL binding sites on 4-1BB are independent of one another (fig. S8).

**Fig. 6.**
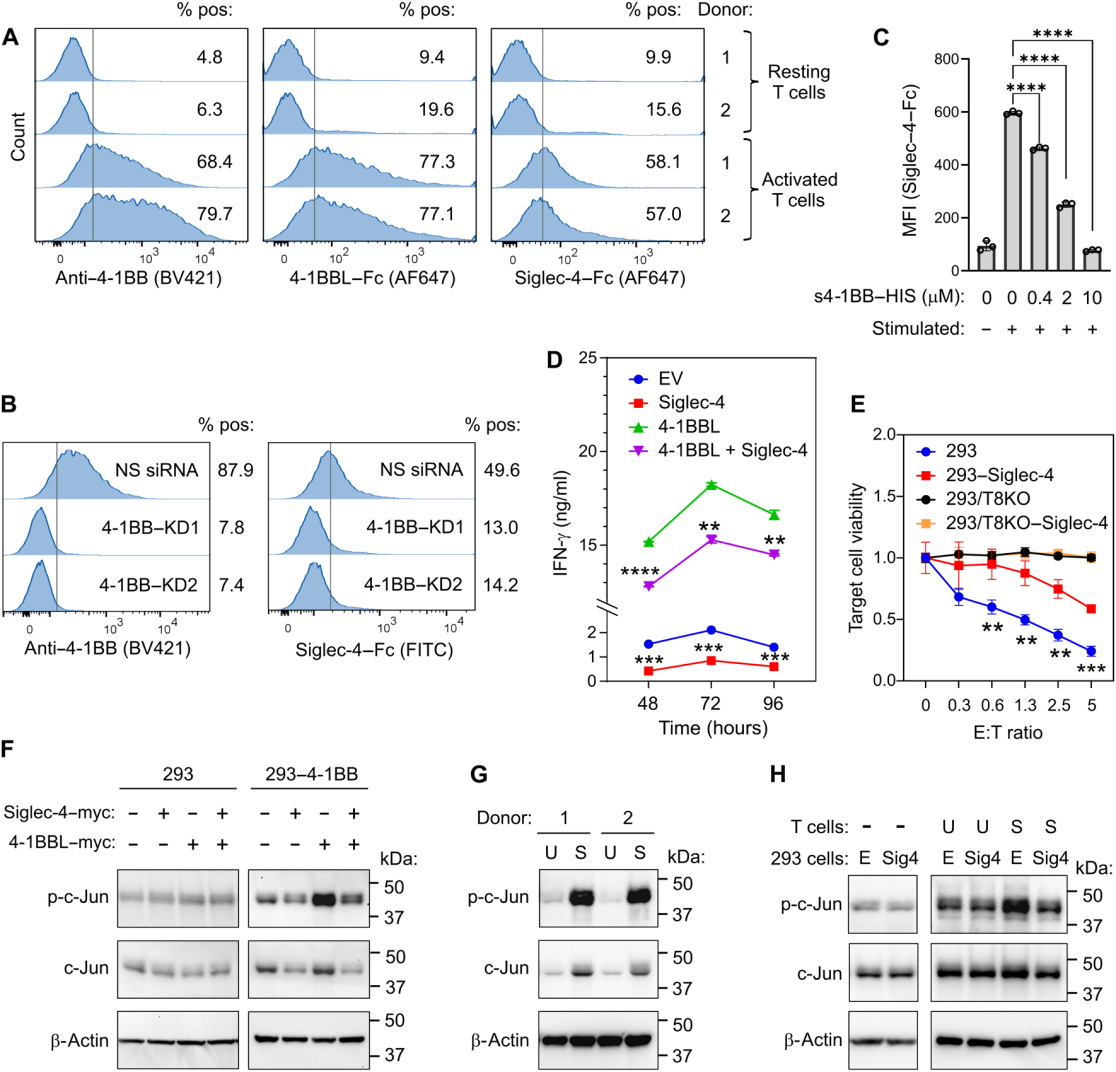
Siglec-4 suppresses 4-1BB–mediated T cell activation. (**A** to **C**) FC was used to monitor (A) 4-1BB expression and 4-1BBL–Fc or siglec-4–Fc binding to activated T cells, (B) binding of siglec-4–Fc to stimulated T cells transfected with nonspecific (NS) or 4-1BB knockdown (KD) siRNAs, or (C) binding of siglec-4–Fc to stimulated T cells in the presence of increasing amounts of soluble 4-1BB–HIS protein. MFI, mean fluorescence intensity. Statistical analysis: unpaired *t* test. (**D**) ELISA was used to measure IFN-γ produced by activated T cells mixed with 293 cells overexpressing EV, siglec-4, 4-1BBL, or siglec-4 plus 4-1BBL. Statistical analysis: unpaired *t* test; *P* values correspond to comparisons between groups with or without siglec-4. (**E**) Luciferase assays were used to measure the viability of eGFP-FFLuc–labeled 293 target cells 24 hours after mixing with anti–TEM8–CAR-T cells. TEM8 knockout control cells (293/T8KO) were included as a specificity control. E:T, effector:target cell ratio. Statistical analysis: unpaired *t* test; *P* values correspond to comparisons between 293 and 293–Siglec-4 at each E:T cell ratio. (**F** to **H**) Immunoblotting was used to assess (F) p-c-Jun and c-Jun levels in 293 cells or 293–4-1BB cells following transient transfection with full-length siglec-4–myc or 4-1BB–myc, (G) p-c-Jun and c-Jun levels in unstimulated (U) or stimulated (S) T cells derived from two independent donors, and (H) p-c-Jun and c-Jun levels in T cells cocultured for 1 hour at a ratio of 1:1 with 293 cell transfected with EV (E) or siglec-4 (Sig4). Note that Siglec-4 expression can mediate the down-regulation of c-Jun only if 4-1BB is also present. β-Actin was used as a loading control in (F), (G), and (H). All data or images in (A) to (H) were representative of at least three independent experiments. For (C) to (E), *n* > = 3 biologically independent samples per group. **P ≤ 0.01, ***P ≤ 0.001, ****P ≤ 0.0001.

To explore the functional consequence of the siglec-4/4-1BB interaction, we began by evaluating the binding of siglec-4 to activated T cells, as 4-1BB is induced in T cells upon activation (*42*). Siglec-4–bound 4-1BB–positive T cells activated by anti-CD3/CD28 treatment, while no binding was observed with resting T cells (Fig. 6A). Knockdown of 4-1BB using small interfering RNA (siRNA) or competition with soluble 4-1BB ED (s4-1BB–HIS) abolished siglec-4 binding to activated T cells, confirming specificity (Fig. 6, B and C). Next, we performed T cell activation and proliferation assays where we labeled activated primary human T cells with the PKH26 dye and then mixed them with 293 cells overexpressing EV, siglec-4, 4-1BBL, or both siglec-4 and 4-1BBL. As shown in fig. S9, siglec-4 expression was unable to potentiate proliferation, whereas 4-1BBL strongly stimulated T cell proliferation and activation. In addition, siglec-4 markedly inhibited 4-1BBL–induced proliferation and interferon-γ (IFN-γ) secretion, without affecting 4-1BBL protein level (Figs. 5D and 6D and fig. S9), indicating its inhibitory effect on T cell activation. To determine whether siglec-4 could affect chimeric antigen receptor T (CAR-T) cell activity, we created CAR-T cells against the cancer-associated cell surface protein Tumor Endothelial Marker 8 (TEM8)/Anthrax toxin receptor 1 (ANTXR1) (fig. S10) and tested them against TEM8^+^ 293 cells with or without siglec-4. 293-TEM8 knockout cells were also included as a specificity control. Overexpression of siglec-4 in 293 cells blocked anti-TEM8 CAR-T killing specifically against TEM8-positive target cells (Fig. 6E). To further understand the downstream signaling pathways affected by siglec-4, a phospho-kinase array was used to evaluate alterations in kinase signaling in 293/4-1BB cells mixed with 293/4-1BBL cells overexpressing either EV or siglec-4. This study revealed that the levels of phospho-c-Jun (p-c-Jun), a transcription factor recently found to be critical for optimal CAR-T cell activation (*43*), decreased markedly in the presence of siglec-4 (fig. S11). Further analysis in 293 cells using Western blotting revealed that the both p-c-Jun and total c-Jun protein levels decreased following siglec-4 expression but only with the presence of 4-1BB (Fig. 6F), indicating that the inhibitory effect of siglec-4 on c-Jun depends on 4-1BB. As expected, p-c-Jun was induced in activated T cells (Fig. 6G) but decreased by coculturing activated T cells with 293 cells overexpressing siglec-4 but not EV, partially due to the down-regulation of c-Jun by siglec-4 (Fig. 6H). Together, these results indicate that in trans binding to siglec-4^+^ cells may block 4-1BB–mediated T cell activation through inhibition of c-Jun signaling.

## DISCUSSION

Transmembrane receptors and their ligands are key targets for drug development, and deorphanizing unknown receptor-ligand interactions holds tremendous promise for future drug discovery. Current approaches for deorphanizing receptors face challenges due to the amphipathic nature of transmembrane proteins and high costs. To help address these limitations, a cell-based pooled CRISPRa screening strategy was introduced by the Wright laboratory in 2018 (*15*). However, we found that low-affinity (micromolar *K*_D_) ligand-receptor pairs were not readily identified using this approach. To overcome this issue, we explored methods to improve binding affinity and increase the probability of identifying weak transient physiological interactors. Magnetic beads were demonstrated to enrich interactions with micromolar affinity, while the original approach typically detects submicromolar interactions. Our findings revealed a low-affinity interaction between 4-1BB and siglec-4. Further investigation demonstrated that siglec-4 could suppress 4-1BB–mediated T cell activation, indicating an immunosuppressive role that may act to regulate the immunostimulatory role of 4-1BB. Although further studies are required to fully understand the function of siglec-4/4-1BB interaction in immune system, these data potentially help to explain the paradoxical roles of 4-1BB in autoimmunity (*35*–*38*).

Baited magnetic beads not only improved enrichment sensitivity but also enabled an evaluation of enrichment kinetics. FC enrichment monitoring using fluorescently labeled bait-bound beads allowed us to determine whether the screening campaign was a success before sequencing. On the basis of our experience, we speculate that it may only be necessary to sequence the library if a clear enrichment is observed by FC monitoring following serial sorting, potentially eliminating the need for extensive sequencing. Also, the FC monitoring allows researchers to decide when to stop the serial enrichment, which could vary by gene depending on the number of functional guides present in the starting library. Last, if a large fraction of positive cells were detected by FC monitoring, then it is possible to identify the main target with fewer sequencing reads. Because sequencing was the most expensive aspect of the original CRISPRa screening approach, avidity-based enrichment helps make CRISPRa more affordable.

Advances in guide design offer another strategy to improve the CRISPRa screening approach. For this, we created a library with a limited number of highly selective guides using more stringent criteria to minimize the number of “off-target” hits and reduce sequencing costs. While working with our PD-L1 bait, we observed a faster enrichment of *PDCD1* guides using the new library. However, in some cases, the new library showed slower amplification than the original one. On the basis of this, we infer that, for some screening campaigns, higher guide numbers per gene may be more important than stringent guide selection. To optimize cost and success probability, researchers can start with the smaller, stringent library and proceed to sequencing only if clear amplification is observed by FC monitoring. If FC enrichment is not observed, then one could then redo the selection with the larger original library. Alternatively, combining the two libraries from the outset can provide a strong probability of success in the shortest period of time.

While high-avidity screening has advantages in deorphanizing receptor-ligand pairs, the approach still has some limitations. Baits that bind obligately to complexes involving multiple proteins may not be uncovered using this approach. Furthermore, if the activator cells highly express the target binding partner endogenously, then it may not be easy to enrich through guide activation due to the competition. However, this potential problem can be identified by initially screening activator cells without library transduction and overcome by using alternative activator cell lines that do not bind to the bead-bound bait. Another issue is the enrichment of certain guides (e.g., *SDC1* and *SDC2*) by multiple baits, which may relate to the promiscuous binding of the encoded proteins (*28*). Fortunately, the streptavidin beads did not share this problem, providing a possible solution and suggesting that syndecan binding may relate to the particular combination of beads and baits used for the selection. Last, while purified recombinant HIS- and Fc-ED fusions for many surface proteins are commercially available and ready for immediate use with protein A and anti-HIS beads, fusion proteins containing a C-terminal biotin tag for streptavidin bead conjugation may require added time for in-house production and purification.

In summary, our high-avidity screening platform enables the deorphanization of receptor-ligand pairs with high sensitivity. Since weak protein interactions at the cell surface are the most difficult to unravel, many of the remaining orphan ligand-receptor interactions are likely to have low affinity. On the basis of the successes of targeting receptor-ligand interactions in the clinic, deorphanizing such interactions should ultimately aid in efforts to create next-generation therapeutics.

## MATERIALS AND METHODS

### Cell lines and culture

HEK293 (American Type Culture Collection, CRL-3216) and LentiX-293 T (Takara, cat. no. 632180) cells were maintained in Dulbecco’s modified Eagle’s medium (DMEM) supplemented with 10% fetal bovine serum (FBS) and passaged every 2 to 3 days. Suspension and serum-free adapted Freestyle 293-F cells (Thermo Fisher Scientific, cat. no. R79007) were routinely cultured in Freestyle 293 expression media (Thermo Fisher Scientific, cat. no. 12338018) and maintained in 37°C incubators with shaking (125 rpm) and passaged every 2 to 3 days. Untransduced T cells or CAR-T cells were maintained in RPMI 1640 medium supplemented with 10% FBS and recombinant human interleukin-2 (IL-2; 50 IU/ml; Teceleukin, Biological Resources Branch, NCI-Frederick) and passaged every 2 to 3 days.

### Plasmids

To produce Fc fusion protein vectors, the ED of CTLA4 (Met^1^-Phe^162^, GenBank no. NP_005205) or PD-L1 (Met^1^-Arg^238^, GenBank no. NP_054862) was fused in frame with human Fc (Asp^103^-Lys^329^, GenBank no. AAC82527) and cloned into the pcDNA3.1^+^ vector (Thermo Fisher Scientific, cat. no. V79020). Fc fusion proteins were also engineered to contain the L234A, L235A, and P329G mutations (*44*) to prevent binding to Fcγ receptors. To generate biotinylated bait proteins, the EDs of human CTLA4 (Met^38^-Phe^162^, NP_005205), PD-L1 (Met^1^-Arg^238^, GenBank no. NP_054862), and 4-1BB (Met^1^-Ser^189^, GenBank NP_001552) were cloned between the Nhe I and Sbf I restriction enzyme sites of the CD4d3 + 4-bio-HIS plasmid (Addgene plasmid no. 36153) and then cotransfected with a BirA-Flag plasmid expressing biotin ligase developed by G. Wright’s laboratory (Addgene, plasmid no. 64395). For the biotinylated CTLA4 ED, the endogenous signal peptide was replaced with a signal peptide from human immunoglobulin G (IgG). All constructs were sequence-verified.

### gRNA library preparation

The human membrane protein activation library developed by G. Wright was obtained from Addgene (cat. no. 113345). The library DNA was electroporated into NEB 10-beta electrocompetent *E. coli* [New England Biolabs (NEB), cat. no. C3020K] at 2.0 kV, 200 Omega and 25 μF using a Gene Pulser Xcell Electroporation System (Bio-Rad). Transformed bacteria were then grown in 500 ml of 2× Yeast Extract Tryptone (YT) medium/amp (ampicillin; 50 μg/ml) medium and incubated at 37°C overnight with shaking at 220*g*. A Qiagen plasmid maxi kit (Qiagen, cat. no. 12163) was used to purify library DNA from 500 ml of bacteria culture following the manufacturer’s instruction. To determine the library representation after purification, 10 ng of purified plasmid DNA was used as template for polymerase chain reaction (PCR) amplification and sequencing using the Illumina MiSeq.

### Individual gRNA cloning

The gRNAs *WNT7A-6*, *TSPAN17-4*, *ICA1-4*, *BNIP3L-2*, *PDCD1-1/2/3/4/5/6/7*, *CD86-1/2/3/4/5/6/7*, *CD80-2/3/4/5/13/14*, *siglec-4-2/4/6*, and *TNFSF9-1/2/3/4* (*15*) were synthesized from Integrated DNA Technologies as 25-bp oligomers and cloned into the pKVL2-U6gRNA_SAM(BbsI)-PGKpuroBFP-W plasmid from K. Yusa (Addgene, plasmid no. 112925) by digesting the vector with Bbs I for 2 hours at 37°C, followed by gel purification. The synthesized gRNA oligomers were 5′ phosphorylated with T4 Polynucleotide Kinase (NEB, cat. no. M0201S) in 1× T4-ligation buffer (NEB, cat. no. B0202S) for 30 min at 37°C followed by annealing at 95°C for 5 min before slowly decreasing the temperature to 25°C at 5°C/min. Annealed oligos were ligated into the digested vector by incubating with Quick Ligase (NEB, cat. no. M2200S) for 10 min at room temperature (RT). Cloned vectors were sequence-verified.

### Lentivirus production

For lentivirus packaging, 1 day before transfection (day −1), 6 × 10^6^ LentiX-293 T cells were seeded and cultured in a 100-mm dish containing 10 ml of DMEM complete media (DMEM supplemented with high glucose and sodium pyruvate, 10% FBS, and penicillin/streptomycin). The next day (day 0), cell media were exchanged with 10 ml of fresh media without antibiotics, and cells were cotransfected with 3 μg of gRNA plasmid, 2 μg of pMD2.G lentiviral VSV-G envelope expressing plasmid, and 5 μg of psPAX2 packaging plasmid (Addgene, plasmid nos. 12259 and 12260) using 40 μl of lipofectamine 2000 (Thermo Fisher Scientific, cat. no. 11668019) diluted in 500 μl of Opti-MEM transfection media. Six hours later, media were replaced with 10 ml of fresh complete media. The viral supernatant was collected at days 1, 2, and 3. The combined supernatants were spun down at 1000*g* for 10 min and filtered using a 0.45 filter. Filtered virus was then concentrated using a Lenti-X Concentrator (Takara, cat. no. 631232) according to the manufacturer’s instruction, aliquoted, and stored at −80°C.

### Lentivirus titration and transduction

Lentivirus titers were determined by transducing HEK293 cells with a serial dilution of virus and quantifying the percentage of blue fluorescent protein (BFP)–positive cells 48 hours posttransduction using FC. Briefly, HEK293 cells were seeded in six-well plates (5 × 10^5^ cells per well) and incubated overnight under normal culture conditions. The next day, one aliquoted vial of concentrated virus stock was thawed on ice and titrated using media containing hexadimethrine bromide (polybrene) (8 to 10 μg/ml; Sigma-Aldrich, cat. no. H9268). In duplicate, 10-fold serial dilutions of the virus solution were prepared from 1 × 10^−1^ to 1 × 10^−5^ in polybrene-containing complete media changing pipet tips between each dilution and mixing well to ensure a homogeneous solution. Diluted virus was then used to replace media in each six-well well. Nontransduced cells were used as a negative control. At day 2, media were replenished with complete media. Cells were monitored and analyzed for BFP expression at day 3 using FC to calculate BFP-positive cells and virus titer according to the equation: TU/ml = (initial cell count × % BFP positive)/dilution.

### Activator cell line establishment

To establish a stable activator cell line overexpressing the VP64-dCas9-VP64 and MS2-p65-HSF1 fusion proteins, HEK293 cells were cotransfected with pPB-R1R2_EF1aVP64dCas9VP64_T2A_MS2p65HSF1-IRESbsdpA deposited to Addgene by G. Wright’s laboratory (Addgene, plasmid no. 113341) (*15*) and Super PiggyBac Transposase expression vector (System Biosciences, cat. no. PB210PA-1) in a 5:1 ratio. Forty-eight hours posttransfection, cells were selected with at blasticidin S HCL (5 μg/ml; Thermo Fisher Scientific, cat. no. A1113903), and fresh media with blasticidin were replenished every 2 to 3 days. After 2 weeks, live cells were cloned by limiting dilution into 96-well plates. The clone with the highest CRISPRa activity was identified using a CRISPR reporter system. For this, different activator cell clones were cotransfected with a plasmid carrying an AAVS1 gRNA (GTCCCCTCCACCCCACAGTG, pTS0057), a reporter plasmid containing an AAVS1 target site within a minCMV reporter driving tdTomato (reporter-gT1, Addgene, plasmid no. 47320), and pAcGFP1-N1 (Takara, cat. no. 632469) at 1:2:1 ratio, using the pAcGFP1-N1 constitutive green fluorescent protein (GFP) vector as an internal indicator of successful transfection. pTS0057 was generated by ligating corresponding oligonucleotides into the digested lenti sgRNA(MS2)_puro backbone (Addgene, plasmid no. 73797) and was deposited into Addgene under #200832. HEK293 cells were used as control. GFP/Tdtomato expression was analyzed at 48 hours posttransfection by FC on a BD LSRFortessa flow cytometer (BD Biosciences) to measure and compare activation efficiency of different clones. The final activator cell clone used was called 293-VM-14.7, where VM stands for dCas9 with VP64 and MS2p65HSF1, and 14.7 represents the subclone with the highest CRISPR activity in the reporter assay.

For library transduction, to ensure adequate gRNA representation, i.e., at least 200× coverage, 5 × 10^7^ 293-VM-14.7 cells were transduced with lentivirus carrying gRNA library at an MOI of 0.3 to assure that most transduced cells only received one viral particle, which was confirmed by the presence of around 30% BFP-positive cells before drug selection. Forty-eight hours posttransduction, transduced cells expressing the gRNA library were selected with puromycin (2 μg/ml; InvivoGen, cat. no. ant-pr-1), and BFP was used to monitor the percent positive cells. After 2 weeks’ maintenance, most cells were BFP positive and ready for subsequent screening. Library-transduced cells were maintained in DMEM complete media containing puromycin (0.5 μg/ml) and passaged every 2 to 3 days. To ensure at least 300 to 500× coverage for the gRNA library selection, 2 to 3.5 × 10^7^ of cell stock was used for each selection.

### Recombinant protein expression and purification

To generate biotin-labeled baits, ED-CD4d3 + 4-bio-HIS plasmids containing a C-terminal biotinylation peptide were cotransfected with secreted BirA plasmid encoding biotin ligase at a ratio of 9:1 into Freestyle 293-F cells using 293fectin (Thermo Fisher Scientific, cat. no. 12347019) according to the manufacturer’s instruction. pcDNA3.1^+^ plasmids encoding Fc-tagged proteins were also transfected into Freestyle 293-F cells using 293fectin. Transfected Freestyle 293-F cells were maintained in Freestyle 293 expression medium (Thermo Fisher Scientific, cat. no. 12338018) with shaking at 37°C, 125 rpm, for 7 days. Next, supernatants were collected and filtered through a 0.45-μm filter bottle, followed by purification with Ni Sepharose 6 Fast Flow (VWR International, cat. no. 97067-844) for biotinylated proteins and Pierce Protein G Agarose beads (Thermo Fisher Scientific, cat. no. 20397) for Fc-tagged proteins in accordance with the manufacturer’s instructions and then dialyzed into phosphate-buffered saline (PBS) using Slide-A-Lyzer Dialysis Cassettes (Thermo Fisher Scientific, cat. no. 66330). For HIS-protein purification, dialysis in PBS using SnakeSkin Dialysis Tubing (Thermo Fisher Scientific, cat. no. 68100) was performed before Ni Sepharose purification.

### Tetramerization of biotinylated proteins

To determine how much biotinylated bait was needed to saturate a fixed amount of AF488-streptavidin (Thermo Fisher Scientific, cat. no. S11223), different amounts of biotinylated bait proteins were incubated with AF488-streptavidin at 4°C overnight, and then complexes were purified by size exclusion chromatography (SEC) using an Amersham Biosciences AKTA FPLC System (Amersham). Fractions with the largest size, representing the largest complex with the most bait proteins, were collected and used for cell staining and sorting.

### Fluorescence-activated cell sorting

To test the original selection system from Wright’s laboratory, 1.25 × 10^8^ library-transduced activator cells were incubated with 125 μg of SEC-purified biotinylated bait complexed with AF488-streptavidin in PBS/1% bovine serum albumin (BSA) with rotation at 4°C for 2 hours. Stained cells were washed, collected, and resuspended in PBS/1% BSA and sorted using a BD FACSAria III cell sorter. After gating BFP-positive cells, the top 5% FITC-positive cells were collected.

### Bait protein–magnetic bead conjugation

Streptavidin microbeads, protein A microbeads, and anti-HIS microbeads (Miltenyi Biotec, cat. nos. 130-048-101, 130-071-001, and 130-091-124) were conjugated with biotinylated, Fc-tagged, and HIS-tagged bait proteins, respectively. To saturate magnetic beads, for each round of selection, 125 μl of Miltenyi magnetic microbeads was mixed with 30 μg of specific bait proteins in a 2-ml microfuge tube, using 1 ml of PBS, and tubes were rotated at 4°C overnight. One day later, excess unbound protein was removed using MS columns (Miltenyi Biotec, cat. no. 130-042-201). Briefly, 0.5 ml of sterile balance buffer (PBS/0.5%BSA, pH = 7.4) was used to equilibrate the column, followed by loading of the protein-microbead solution. Then, columns were washed three times with 0.5 ml of balance buffer and the protein-microbead complexes were eluted in 1 ml of balance buffer using the plunger. The ready-to-use bait protein-microbead complexes were stored at 4°C for up to 2 weeks.

### Magnetic-activated cell sorting

To ensure a sufficient library coverage, 2 to 3.5 × 10^7^ activator cells were used to initiate MACS. When activator cells and protein-microbead complexes were both ready, library-transduced cells were detached using Accutase cell detachment solution (Corning, cat. no. 25-058-CI), and cells were washed once with balance buffer. Then, the cells were resuspended in ready-to-use cold bait protein-microbead complexes on ice, followed by rotating at 4°C for 1.5 hours before performing MACS using the QuadroMACS separator over an LS column (Miltenyi Biotec, cat. nos. 130-090-976 and 130-042-401). For MACS, the column was balanced with 3 ml of balance buffer before loading 1 ml of cell suspension by pipetting 0.5 ml at a time. Immediately after all cells passed through the column, balance buffer was used to wash the column. For the first and second rounds of MACS, three rinses with 0.5 ml of buffer were used to wash the column (1.5 ml in total) to minimize the early loss of cells with a low-binding affinity; in later rounds, the column was washed six times with 0.5 ml of buffer (3 ml in total). After washing, the column was removed from the separator and placed on a 15-ml conical tube. Five milliliters of balance buffer was then pipetted onto the column, and the magnetically labeled cells were immediately flushed out by firmly pushing the plunger into the column. Eluted cells were collected and cultured in DMEM complete media. Once cell numbers reached 8 × 10^7^, which typically occurred sooner with each successive selection and ranged between 1 and 3 weeks, 5 × 10^6^ cells were collected to extract gDNA, 5 × 10^6^ cells were stained for enrichment monitoring, 3.5 × 10^7^ cells were frozen in 90% FBS/10% dimethyl sulfoxide, and 3.5 × 10^7^ cells were used to conduct the next-round of MACS if needed.

### FITC labeling of microbeads

While streptavidin beads could potentially be used for enrichment monitoring, our initial attempts to colabel streptavidin beads with biotin-labeled bait proteins and FITC-biotin failed, prompting us to test protein A beads instead. Protein A microbeads were dual labeled with FITC and bait protein using a previously optimized protocol (*45*). Briefly, for each sample labeling, 10 μl of protein A microbeads was incubated with 0.8 μg of Fc-tagged bait proteins and 0.2 μg of Fc-FITC (Novus, cat. no. NBP1-97252). PBS was then added to reach a volume of 100 μl before rotating the mix for 1 hour at RT or overnight at 4°C.

### Flow cytometry

For antibody-based FC staining of CD80, CD86, PD-1, 4-1BBL, 4-1BB, syndecan-1 (SDC-1), or syndecan-2 (SDC2), FITC-anti-human CD80 (Thermo Fisher Scientific, cat. no. 11-0809-41), Allophycocyanin (APC)–anti-human CD86 (BioLegend, cat. no. 305411), AF488-anti-human CD86 (BioLegend, cat. no. 305413), AF488-anti-human PD-1 antibody (BioLegend, cat. no. 329936), APC-anti–h4-1BBL antibody (BioLegend, cat. no. 311506), phycoerythrin (PE)–anti–h4-1BB antibody (BioLegend, cat. no. 300804), Brilliant Violet (BV) 421 anti–h4-1BB antibody (BioLegend, cat. no. 309819), PE-anti-human SDC1 (Thermo Fisher Scientific, cat. no. 12-1389-41), or PE-anti-human SDC2 (R&D, cat. no. FAB2965P) was used. For one-layer staining, 0.2 μg of specific fluorescence-conjugated primary antibodies was used to stain 5 × 10^5^ cells in 100 μl of PBS/0.5% BSA (PBS/BSA). For cell surface siglec-4 protein detection, 0.5 μg anti-human siglec-4 antibody (BioLegend, cat. no. 851702) was used followed by AF594 AffiniPure Donkey Anti-Mouse IgG (H + L) (Jackson ImmunoResearch, cat. no. 715-585-150) or AF647 AffiniPure Donkey Anti-Mouse IgG (H + L) (Jackson ImmunoResearch, cat. no. 715-605-150) to stain 5 × 10^5^ cells in 100 μl of PBS/BSA. To assess TEM8 levels in 293 TEM8 wild-type and knockout cell lines, cells were stained with m825 anti-TEM8 antibody followed by FITC-Donkey Anti-Human IgG (Jackson ImmunoResearch, cat. no. 709-095-149). After staining, cells were washed twice with PBS/BSA before FC analysis. Samples were then fixed in 1% paraformaldehyde and analyzed on a BD LSRFortessa flow cytometer (BD Biosciences). FC data were generated from at least 10,000 cells per sample after gating. Because variation between replicate samples processed at the same time was negligible, most data presented were derived from representative experiments that were repeated on separate days, as indicated in the figure legends. The data were analyzed and processed using FlowJo software (BD Biosciences).

For FC monitoring of enrichment following each round of MACS using bait protein as a probe, 5 × 10^5^ sorted cells were incubated with 100 μl of FITC-Fc-protein A microbeads-Fc bait protein complex (prepared as described above under the “FITC labeling of microbeads” section) and rotated for 1 hour at 4°C. Activator cells before MACS enrichment were also stained as a negative control. For staining cells with FITC-labeled bait-bound protein A microbeads, FITC-labeled protein A microbeads without bait were also used as a staining control. Before performing FC analysis, cells were rinsed twice with PBS/BSA.

For h4-1BB protein binding assay, HEK293 cells were transfected with pCMV6 (Origene, cat. no. PS100001) or pCMV3 (SinoBiological, cat. no. CV011) EV controls, pCMV-human siglec-4 (Origene, cat. no. RC208754), or pCMV-h4-1BBL (SinoBiological, cat. no. HG15693-CM) plasmids using Lipofectamine 2000 transfection reagent (Thermo Fisher Scientific, cat. no. 11668019) according to the manufacturer’s manual. Forty-eight hours posttransfection, 5 × 10^5^ transfected cells were incubated in 100 μl of PBS/0.5% BSA with 1 μg of h4-1BB–HIS protein on ice for 30 min, rinsed with PBS/BSA, stained with 1 μl of PE-anti-HIS (Miltenyi Biotec, cat. no. 130-120-718) on ice for 15 min, rinsed with PBS/BSA, and then analyzed by FC. For the m4-1BB protein binding assay, HEK293 cells transfected with EV, pCMV–m-siglec-4 (SinoBiological, cat. no. MG51398-CM) or pCMV–m4-1BBL (SinoBiological, cat. no. MG50067-CM) were incubated with 1 μg of m4-1BB–Fc protein (SinoBiological, cat. no. 50811-M02H) followed by staining with AF647 AffiniPure Donkey Anti-Human IgG (H + L) (Jackson ImmunoResearch, cat. no. 709-605-149).

For the syndecan binding studies, HEK293 activator cells transfected with EV, human SDC1 (SinoBiological, cat. no. HG11429-UT), human SDC2 (SinoBiological, cat. no. HG10355-CF), or a mixture of plasmids composed of the most highly enriched individual *CD80-3/4/5/13* gRNAs were stained with 1 μg of biotinylated CTLA4 protein/5 × 10^5^ cells in 100 μl of PBS/BSA followed by APC-streptavidin staining.

To monitor binding of siglec-4 ED to resting and activated T cells, T cells were preincubated with human BD Fc Block (BD Biosciences, cat. no. 564220) for 10 min at RT followed by staining with h4-1BBL–Fc protein (SinoBiological, cat. no. 15693-H01H), or human siglec-4–Fc protein (R&D, cat. no. 8940-MG-050), and subsequent detection using AF647 AffiniPure Donkey Anti-Human IgG (H + L) (Jackson ImmunoResearch, cat. no. 709-605-149).

### Genomic DNA extraction and deep sequencing

Guided by the original method (*15*), the top 5% FITC-positive cells sorted by FACS, around 1 × 10^6^ cells, were collected for gDNA extraction. For the avidity-enhanced protocol described here, 5 × 10^6^ cells from the MACS enrichment were washed once with PBS before extracting gDNA using the DNeasy Blood and Tissue kit (Qiagen, cat. no. 69506) according to the manufacturer’s protocol with some modifications: Briefly, cells were collected into 1.5-ml microcentrifuge tubes, pelleted at 300*g* for 5 min, and then resuspended in 200 μl of fresh PBS and sequentially mixed with 200 μl of AL buffer and 20 μl of protease K. Samples were immediately incubated on a thermomixer (Eppendorf, EP5382000015) at 56°C, 1450 rpm for 10 min. Samples were precipitated by adding 200 μl of ethanol (96 to 100%) and vortexing for 10 to 15 s and purified in the DNeasy Mini spin column, rinsing with 500 μl of buffer AW1 and then 500 μl of buffer AW2, and eluting with 200 μl of buffer AE. As a starting control for comparison, gDNA from 2.5 × 10^7^ library-transduced nonsorted cells was used. The gDNA concentration was quantified with a Qubit Fluorometer (Invitrogen, cat. no. Q33226). The libraries used for sequencing were prepared from two rounds of PCR. In the first round, gRNA sequence was amplified from gDNA. For individual PCR reactions, 1 μg of gDNA was used in a 50 μl of PCR reaction with Q5 enzyme (Q5 Hot Start High-Fidelity 2X Master Mix, NEB, cat. no. M0494L), 2.5 μl of 10 μM primer mix (primer sequences can be found in table S4), and 0.5 μl of SYBR Green (Thermo Fisher Scientific, cat. no. S7563) prediluted 1/400 with distilled water (dH_2_O). In total, eight PCR reactions were run for each sample in a quantitative PCR (qPCR) machine (98°C for 3 min and then 32 cycles of 98°C 15 s, 61°C 15 s, and 72°C 15 s). The PCR reaction was terminated manually for each individual sample before the PCR amplification curve reached plateau. Then, the PCR product was cleaned and purified using the AMPure XP beads (Beckman Coulter, cat. no. A63882) or the GeneRead Size Selection Kit (Qiagen, cat. no. 180514). The purified PCR product was quantified with a Qubit Fluorometer. For the second round of PCR, Illumina sequencing adaptors were added to the above-purified amplicons (table S4). For each individual PCR reaction, 20 ng of DNA was used in a 20-μl PCR reaction with Q5 enzyme, 1 μl of 10 μM Illumina adaptor mix and 0.2 μl diluted SYBR. In total, eight PCR reactions were run for each sample in a qPCR machine (98°C for 3 min and then 6 cycles of 98°C 15 s, 61°C 15 s, and 72°C 15 s). The PCR reaction was manually terminated for each individual sample before the PCR amplification curve reached plateau. Then, the PCR product was purified using the E-gel system (Thermo Fisher Scientific, cat. no. G401002) and the Zymoclean Gel DNA Recovery Kit (Zymo Research, cat. no. D4002) sequentially. The final purified amplicon libraries were quantified using a Qubit Fluorometer. Appropriate amounts of DNA for each sample, based on the amount of input DNA used, were pooled together into a single tube and were sequenced on either the Illumina NextSeq or NovaSeq platform using the 1 × 100 cycle format.

### Analysis of high-throughput sequencing data

Raw FASTQ files were first trimmed using cutadapt to remove sequences 5′ to the gRNA sequence (for further rcutadapt details, see https://journal.embnet.org/index.php/embnetjournal/article/view/200). Trimmed FASTQ files were then used as input into mageck (*46*). Mageck “test” was used to determine genes which were enriched at statistical significance using a false discovery rate (FDR) threshold of 0.1. The gene enrichment values were calculated by taking the log_10_ of the RRA (robust rank algorithm) score derived from the Mageck output (gene-based analysis). See https://sourceforge.net/p/mageck/wiki/output/ for more details. The Mageck output files for each of the analysis described can be found in tables S5 to S14.

### Generation of an optimized CRISPRa library

Recent improvements in identifying off-target sites (*30*) and optimization of RNA guide design for CRISPR-based activation (*31*) allow for the design of libraries that are smaller in size but with high activity and specificity. To this end, using the genes targeted in the Wright library, a new gRNA library was designed and generated using sequences primarily from the Calabrese A and B libraries as well as hCRISPRa-V2 library (*32*) with prioritization to those with high specificity. To minimize overall library size and probability of off-target activation, a maximum of five guides per gene were included. In addition, by curating genes from soluble and surface proteome projects (*47*, *48*), we identified several additional secreted genes absent from the original Wright library, which were used to create an add-on library using a similar design approach as described above. In total, 949 additional genes were targeted with 4649 gRNAs. The CRISPR libraries designed here are available from Addgene (cat. no. 207471 for new redesigned library targeting genes curated by the Wright lab, cat. no. 207472 for the new soluble add-on library, and cat. no. 1000000228 for one vial of each). The new libraries contained three sources for CRISPR activation guides: the original Wright library, the hCRISPRa-V2 library, and the Calabrese library. For all of these guides, a Guidescan off-target score was generated. To improve target specificity, the rule set 2 (RS2) score was used as well. Since the Calabrese library was optimized on RS2, when selections were prioritized for activity, the Calabrese (A + B) library was the first source used. The design of this library was done in two iterations using the list of genes identified by the Wright laboratory. First, a highly stringent set of guides (with Guidescan score ≥ 0.2) were selected, and for each gene, they were then ranked by the highest to lowest RS2 score. In this iteration, a number of key genes would have been missed, and as such, a second iteration of gRNA selection was performed prioritizing on RS2. As mentioned earlier, for the genes completely missed or undertargeted by a number of guides, the first source of guides was the Calabrese (A + B) library. Subsequently, guides with the highest RS2 score from the hCRISPRa-V2 and Wright libraries were then selected to round out the library. It should also be noted that some gRNAs were present in two or even all three of the libraries.

Oligonucleotides containing gRNAs and flanking sequence were obtained from Twist Biosciences and subsequently amplified by qPCR using the primers listed (table S4). Double-stranded inserts were then cloned into a backbone carrying the SAM gRNA scaffold as well BFP and puromycin (pRC0610) using NEB HiFi Builder. pRC0610 has been deposited in Addgene under #200834. A total of four reactions with 50 ng of digested backbone with 5 ng of insert were then pooled together and purified using the Zymo Clean and Concentrate 5 and eluted in 12 μl of elution buffer. Five microliters of purified assembly was then electroporated in 25 μl of Lucigen Endura cells and recovered with 2 ml of Super Optimal broth with Catabolite repression (SOC) medium at 30°C. The entire recovery was then plated on 10- to 15-cm agar plates, grown at 30°C overnight. Agar plates were then scraped, pelleted, and subjected to maxiprep purification (Thermo Fisher Scientific, Gene Jet Endo Free). Plasmid libraries were then subjected to a two-step PCR, and representation was verified using the Illumina MiSeq.

### Enzyme-linked immunosorbent assay

High binding multiwell ELISA microplates (Santa Cruz, cat. no. sc-204463) were coated with 200 ng of human siglec-4–HIS (ProSci, cat. no. 11-288) or PD-L1–bio–HIS proteins at 4°C overnight. After washing the plate with PBS/0.05% Tween 20 (Sigma-Aldrich, cat. no. P9416) using an automatic plate washer (BioTek, Model ELx50, cat. no. 4071000), blocking with PBS/2% BSA at 37°C for 1 hour, and then washing again with PBS/0.05% Tween 20, serial diluted h4-1BB–Fc proteins (R&D, cat. no. 838-4B) using PBS/2% BSA were added for 2 hours at 37°C and detected using horseradish peroxidase (HRP)–AffiniPure Donkey Anti-Human IgG (H + L) (Jackson ImmunoResearch, cat. no. 709-035-149). 2,2′-azino-di-(3-ethylbenzthiazoline sulfonic acid) (ABTS) solution (Millipore Sigma, 11684302001) was used a substrate to detect HRP, and plates were read at 405 nM.

### Surface plasmon resonance

Surface plasmon resonance was used to measure the binding affinity of human/m-siglec-4 to human/m4-1BB on a BIAcore X100 instrument (GE Healthcare). Human siglec-4–Fc protein (R&D, cat. no. 8940-MG-050) or m4-1BB–Fc protein (SinoBiological, cat. no. 50811-M02H) was diluted in 10 mM sodium acetate buffer (pH 5.0) and immobilized on a CM5 biosensor chip using an amine coupling kit. h4-1BB–HIS protein (R&D, 838-4B-100) or m-siglec-4–HIS protein (SinoBiological, cat. no. 51398-M08H) diluted with the running buffer HBS-EP [10 mM Hepes (pH 7.4), 150 mM NaCl, 3 mM EDTA, and 0.05% surfactant P20] was allowed to flow through the cells at concentrations ranging from 18 to 3000 nM. After 10 min of dissociation, the chip was regenerated with 10 mM acetate buffer (pH 4.0). The data were fitted with a 1:2 binding model, and the dissociation rate constant was estimated with BIAevaluation software (Biacore).

### Deglycosylation of 4-1BB protein

Purified biotinylated 4-1BB–bio–HIS protein was deglycosylated using peptide *N*-glycosidase F (PNGase F; NEB, cat. no. P0704LVIAL) according to the manufacturer’s instruction. Briefly, 5 μg of biotinylated 4-1BB–bio–HIS protein was combined with 2 μl of glycobuffer 2 (NEB, cat. no. B3704SVIAL) and H_2_O to make a 20-μl total reaction volume, and then 5 μl of PNGase F was added to the reaction and incubated at 37°C for 4 hours. Then, 2 μl of the mixture was denatured in SDS loading buffer and detected by immunoblotting using HRP-streptavidin antibody (Jackson ImmunoResearch, cat. no. 016-030-084). After confirming the deglycosylation efficiency, FC was used to assess the binding of deglycosylated or nondeglycosylated 4-1BB protein to HEK293 cells overexpressing EV, siglec-4, or 4-1BBL as described above.

### Competition assay

HEK293 cells were transfected with EV, 4-1BBL, or siglec-4 expression plasmids for 48 hours. 4-1BB–HIS protein (0.1 μM) was then incubated with increasing amounts of 4-1BBL–Fc or siglec-4–Fc proteins (0.01 to 1 μM in 100-μl volume of PBS/0.5% BSA buffer) at RT for 30 min. Transfected HEK293 cells were trypsinized and collected, and 5 × 10^5^ cells were incubated with the protein mixtures on ice for 30 min followed by PE-anti-HIS (BioLegend, cat. no. 362603, clone J095G46) staining and FC monitoring to assess the binding of 4-1BB protein to 4-1BBL or siglec-4 on cell surface in the presence of the competitor proteins. To validate that siglec-4 protein binds 4-1BB protein on activated T cells, 0.4 μM siglec-4–Fc protein was incubated with increasing amounts of recombinant soluble 4-1BB–HIS ED protein (0.4 to 10 μM in 100-μl volume of PBS/0.5% BSA buffer) at RT for 30 min. T cells activated by CD3/CD28 treatment were collected, and 5 × 10^5^ cells were incubated with the protein mixtures on ice for 30 min followed by rinsing and detection using AF647 AffiniPure Donkey Anti-Human IgG (H + L) (Jackson ImmunoResearch, cat. no. 709-605-149). FC was used to assess the binding (mean fluorescence intensity) of siglec-4–Fc to activated T cells.

### Peripheral blood mononuclear cell isolation, activation, and proliferation

Peripheral blood mononuclear cells (PBMCs) were isolated by density gradient separation using Lymphoprep (STEMCELL Technologies, cat. no. 07801) from the heparinized blood of healthy donor volunteers participating in NCI-Frederick Research Donor Program. PBMCs (5 × 10^5^) were plated in each well of a nontissue culture-treated 24-well plate (Falcon, cat. no. 351147) that had been precoated with a solution of anti-CD3 (1 μg/ml; clone: OKT3) (Biological Resources Branch, NCI-Frederick) and anti-CD28 (clone: CD28.2) (BioLegend, cat. no. 302934) monoclonal antibodies. Cells were cultured in complete RPMI 1640 media containing 10% FBS and 2 mM l-GlutaMAX (Gibco, cat. no. 35050-061) overnight at 37°C. PKH26 (Sigma-Aldrich, cat. no. PKH26GL) was then used to stain activated PBMCs according to the manufacturer’s instruction. Stained PBMCs (2 × 10^5^) were transferred into a new tissue culture-treated 24-well plate and incubated with 5 × 10^5^ HEK293 cells transfected with either 1:1 mix of pCMV6 EV control (Origene, cat. no. PS100001) plus pCMV3 EV control (SinoBiological, cat. no. CV011) or a 1:1 mix of the plasmids pCMV6–siglec-4 (Origene, cat. no. RC208754) plus pCMV3 EV, pCMV3–4-1BBL (SinoBiological, cat. no. HG15693-CM) plus pCMV6 EV, or pCMV3–4-1BBL plus pCMV6–siglec-4. Ninety-six hours after coculture, FC was used to assess T cell proliferation, where PKH26 dye–labeled cells (PE channel) were used to identify the T cell population in the FSC versus SSC dot plot. Proliferation in this assay is based on a dilution of PKH26 signal, as dye is diluted in half with each cell division. The supernatant was collected each day for IFN-γ detection by using an ELISA MAX Deluxe Set Human IFN-γ kit (BioLegend, cat. no. 430115) according to the manufacturer’s instruction. PBMCs from three different donors were used for the assays.

### Knockdown of 4-1BB in activated T cells

4-1BB siRNAs and control siRNA were synthesized by Horizon (Dharmacon Reagents, cat. nos. J-008105-08-0020 and J-008105-09-0020). Activated PBMCs (1 × 10^6^) were transfected with 300 nM individual 4-1BB siRNA or control siRNA using an Amaxa P3 Primary Cell 4D-Nucleofector X Kit S (Lonza, cat. no. V4XP-3032) according to the manufacturer’s protocol. Transfected PBMCs were plated into a 96-well plate for 72 hours. Afterward, 10^5^ transfected PBMCs were preincubated with 2.5 μg of human BD Fc Block (BD Biosciences, cat. no. 564220) for 10 min at RT and then stained with 1 μg of human siglec-4–Fc protein (R&D systems, cat. no. 8940-MG-050) on ice for 30 min followed by AF647 AffiniPure Donkey Anti-Human IgG (H + L) (Jackson ImmunoResearch, cat. no. 709–605-149). 4-1BB was stained using BV421 anti-CD137 (4-1BB) antibody (BioLegend, cat. no. 309819). 4-1BB expression and siglec-4 protein binding were detected using a LSRFortessa flow cytometer (BD Biosciences).

### CAR-T cell production

To create an anti-TEM8 CAR, a single-chain variable fragment (scFv) from the m825 fully human TEM8 antibody (*49*) was cloned in frame with a human CD8 transmembrane domain, a 4-1BB costimulatory cytosolic endodomain, and a CD3ζ domain into an SFG retroviral vector (*50*). HEK293T cells were used to produce retrovirus as described previously (*51*). Briefly, HEK293T cells were cotransfected with 3.7 μg of the m825 scFv-CAR retroviral vector, 3.7 μg of the Peg-Pam-e plasmid providing MoMLV gag-pol, and 2.5 μg of the RDF plasmid expressing the RD114 envelope (*52*), using the GeneJuice transfection reagent (Sigma-Aldrich, cat. no. 70967). Medium was replaced at 24 and 48 hours posttransfection, and the retroviral supernatant was collected at 48 and 72 hours posttransfection, clarified, filtered using a 0.45-μm filter, and stored in −80°C for future use or used immediately. To generate CAR-T cells, 1 × 10^6^ PBMCs were activated as described above and then cultured in RPMI 1640 complete media containing 10% FBS and 2 mM l-GlutaMAX supplemented with recombinant human IL-2 (50 IU/ml; Teceleukin, Biological Resources Branch, NCI-Frederick) 1 day after CD3/CD28 activation. On day 3, 1 ml of the m825 scFv-CAR retroviral supernatant was added to a nontissue culture-treated 24-well plate precoated with retronectin (7 μg/ml; TAKARA BIO INC., cat. no. T100B) and centrifuged at 2000*g* for 90 min at 32°C. Activated PBMCs (0.2 × 10^6^/ml) resuspended in complete media supplemented with IL-2 (50 U/ml) were then added to the wells and centrifuged at 800*g* for 10 min at 32°C. Transduction efficiency was measured 3 days posttransduction by FC using AF647 AffiniPure Goat Anti-Human IgG, F(ab′)₂ fragment specific (Jackson ImmunoResearch, cat. no. 109-605-097).

### Knockout TEM8 gene from HEK293 cells

To create the TEM8–knockout (KO) vector, two pairs of DNA oligonucleotides (*TEM8*-guide-1.1; 5′- CACCGGATCCTTACTGTCCGCAATC-3′ and *TEM8*-guide-1.2; 5′- AAACGATTGCGGACAGTAAGGATCC-3′; *TEM8*-guide-2.1; 5′- CACCGGCCTGAAAGCCGTCATTCAC-3′ and *TEM8*-guide-2.2; 5′- AAACGTGAATGACGGCTTTCAGGCC-3′) were synthesized (Integrated DNA Technologies) and phosphorylated using T4 Polynucleotide Kinase (NEB, M0201S). Each pair was annealed at 95°C for 5 min, slowly cooled to RT, and ligated into a *BsmBI* (NEB, cat. no. R0580)–digested lentiviral expression vector lentiCRISPRv2-mcherry (Addgene, plasmid #99154) using Quick Ligase (NEB, cat. no. M2200S). HEK293 cells were transfected with lentiCRISPRv2-mcherry-*TEM8* gRNAs1 and 2 using Lipofectamine 2000 (Invitrogen, 11668019) according to the manufacturer’s manual. After 72 hours, cells were stained with m825 anti-TEM8 antibody and sorted by FACS to isolate a pooled population of TEM8-negative cells, with parent cells used for gating. Selected cells were expanded and sorted two more times to ensure a homogeneous negative population.

### CAR-T in vitro killing assay

CAR-T cells were used for in vitro killing assays on day 10 or 11 after transduction. HEK293 cells or HEK293-TEM8 KO cells engineered to stably express eGFP-firefly luciferase (eGFP-FFLuc) fusion protein using a previously described retrovial vector coding for eGFP-FFLuc (*53*) were transfected with pCMV-EV or siglec-4 plasmids for 48 hours. Different HEK293 cells (1 × 10^4^) were cocultured with m825 scFv–CAR-T cells in 96-well white plates (Falcon, cat, no. 353296) at different effector:target (E:T) ratios. Triplate wells were plated for each cell group. Twenty-four hours after coculture, d-Luciferin (15 μg/ml) (GoldBio, cat. no. LUCK-1 g) was added to each well, and plates were read at 560 nm using a CLARIOStar microplate reader (BMG Labtech).

### Stable cell lines

HEK293 cells were transfected with pCMV–4-1BBL (Sino Biological, cat. no. HG15693-CM) or pCMV–4-1BB (Sino Biological, cat. no. HG10041-CF) plasmids. Starting 48 hours posttransfection, cells were selected for hygromycin (200 μg/ml) resistance for 1 week and then subjected to FACS using APC–anti–h4-1BBL antibody (Biolegend, cat. no. 311506) or PE–anti–h4-1BB antibody (BioLegend, cat. no. 300804) to isolate 4-1BBL– or 4-1BB–positive cells. HEK293 parent cells were used for gating.

### Human proteome array

HEK293–4-1BBL stable cells (8 × 10^6^) transfected with pCMV-EV or pCMV–siglec-4 plasmids were cocultured with 4 × 10^6^ HEK293–4-1BB stable cells for 1 hour at 37°C. Then, cells were collected for analysis using the Proteome Profiler Human Phospho-Kinase Array Kit (R&D, cat. no. APY003C) according to the manufacturer’s recommended protocol.

### Immunoblotting

To confirm the biotinylation of bait proteins, SEC-purified proteins were loaded and separated on SDS–polyacrylamide gel electrophoresis (PAGE) gels, transferred to a polyvinylidene difluoride (PVDF) membrane, and probed with HRP-streptavidin (Jackson ImmunoResearch, cat. no. 016-030-084). For individual gRNA evaluation, 293-VM-14.7 cells were transduced with lentiviruses carrying individual gRNA (*CD80-2/3/4/5/14/14, CD86–1/2/3/4/5/6/7*, *PDCD1-1/2/*3/*4/5/6/7*, *WNT7A-6, TSPAN17-4*, *ICA1-4*, *BNIP3L-2*, *siglec4-2/4/6*, or *TNFSF9-1/2/3/4*) for 48 hours, lysed in lysis buffer (50 mM tris, 75 mM NaCl, and 1% Triton X-100), and then separated by SDS-PAGE and transferred to a PVDF membrane. Immunoblots were probed with mouse anti-human CD279 (PD-1) antibody (BioLegend, cat. no. 329902), mouse anti–siglec-4 antibody (BioLegend, cat. no. 851702), or mouse anti-human CD137L (4-1BBL) antibody (BioLegend, cat. no. 311502), followed by HRP-conjugated anti-mouse secondary antibody (Jackson Immunoresearch, cat. no. 715-035-151). To detect exogenous myc-tagged siglec-4 and 4-1BBL, transfected HEK293 cell lysates were separated by SDS-PAGE, transferred to PVDF, and probed with HRP–anti–c-Myc mAb (GenScript, cat. no. A00863). For phospho-c-JUN or c-JUN detection, HEK293 and T cell lysates were separated by SDS-PAGE and transferred to PVDF membranes, which were then probed with rabbit anti–phospho-c-JUN (Ser^63^) antibody (Cell Signaling, cat. no. 9261) or rabbit anti–c-JUN (60A8) mAb (Cell Signaling, cat. no. 9165) followed by HRP-conjugated anti-rabbit secondary antibody (Jackson Immunoresearch, cat. no.111-035-003). β-Actin was detected using HRP–anti–β-actin (Santa Cruz, cat. no. sc-47778 HRP). Biotinylated bait proteins, siglec-4, 4-1BBL, phosphorylated c-Jun, c-Jun, and β-actin were visualized using the Pierce SuperSignal West Dura Substrate (Thermo Fisher Scientific, cat. no. 34076) according to the supplier’s instructions and developed using a GeneGnome ECL processor (Syngene).

### In trans immunoprecipitation

HEK293 cells were transiently transfected with pCMV–siglec-4–myc, pCMV–4-1BBL–myc (Sino Biological, cat. no. HG15693-CM), or pCMV–4-1BB–FLAG (Sino Biological, cat. no. HG10041-CF) plasmids for 6 hours, trypsinized, and collected by centrifuging at 400*g* for 5 min. 4-1BB–transfected cells were then cocultured at a ratio of 1:1 with siglec-4– or 4-1BBL–transfected cells. Forty-eight hours later, cells were washed three times with cold PBS and then lysed in lysis buffer (50 mM tris, 75 mM NaCl, and 1% Triton X-100) containing complete protease inhibitor cocktail (Roche, cat. no. 11873580001) for 30 min on ice. Subsequently, cell lysates were scraped using a cell scraper (Biologix, cat. no. SKU 70-1180), collected and clarified by centrifuging at 20,000*g* for 10 min, and then incubated overnight with either anti–c-Myc mAb (Roche, cat. no. 11667149001) or mouse IgG isotype control (Southern Biotech, cat. no. 0107-01). Lysates were immunoprecipitated using protein G agarose beads at RT for 1 hour, separated by SDS-PAGE, and transferred onto a PVDF membrane, which was probed with either HRP–anti–c-Myc mAb (GenScript, cat. no. A00863) or HRP-anti-Flag mAb (Sigma-Aldrich, cat. no. A8592).

### Statistical analysis

A Students *t* test was used to calculate differences between groups in Fig. 6 (C to E). All measurements were taken from distinct samples. Differences between two groups were presented as the mean ± SD. All tests were two-sided, and *P* values < 0.05 were considered statistically significant. To determine genes significantly enriched by *P* value, the Mageck algorithm uses a permutation test where the number of mutations is calculated by multiplying the number of genes in the library by 100, and gRNA sequences are randomly assigned during each permutation (*46*). The FDR (or *q* value) is then calculated by applying the Benjamini-Hochberg multiple hypothesis testing correction on the *P* values generated by the permutation test.
