# Supplementary material for: Uncovering receptor-ligand interactions using a high-avidity CRISPR activation screening platform
Source: Sci Adv. 2024 Feb 14;10(7):eadj2445. doi: 10.1126/sciadv.adj2445 (PMC10866537; doi:10.1126/sciadv.adj2445)
Supplement: Supplementary file 1 — Figs. S1 to S11 Legends for tables S1 to S14 [file sciadv.adj2445_sm.pdf]

Science Advances
AAAS

Supplementary Materials for

Uncovering receptor-ligand interactions using a high-avidity CRISPR activation screening platform

Liping Yang *et al.*

Corresponding author: Brad St. Croix, stcroixb@mail.nih.gov; Raj Chari, raj.chari@nih.gov

Sci. Adv. **10**, eadj2445 (2024)
DOI: 10.1126/sciadv.adj2445

The PDF file includes:

Figs. S1 to S11
Legends for tables S1 to S14

Other Supplementary Material for this manuscript includes the following:

Tables S1 to S14

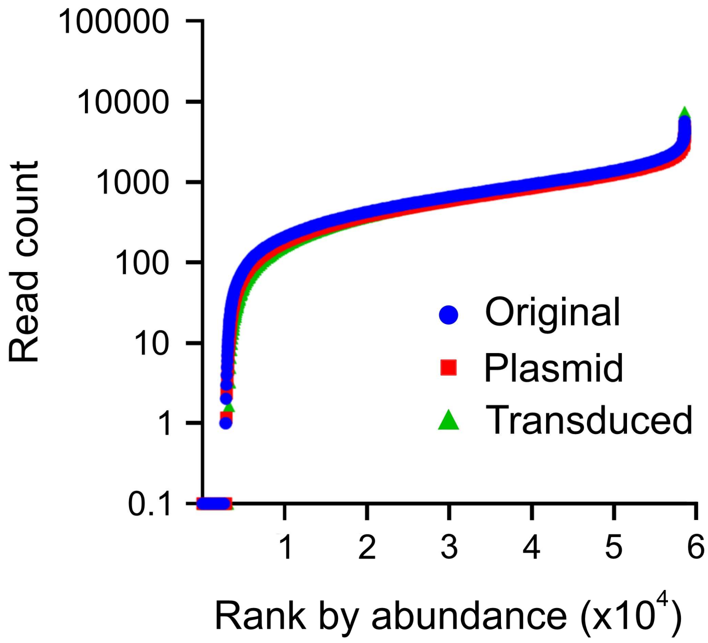

Fig. S1. Library diversity is preserved in 293-VM-14.7 activator cells. Deep sequencing was used to verify that the library maintained its diversity following amplification in *e.coli* (Plasmid) and following puromycin selection of gRNA library virus-transduced 293-VM-14.7 activator cells (Transduced).

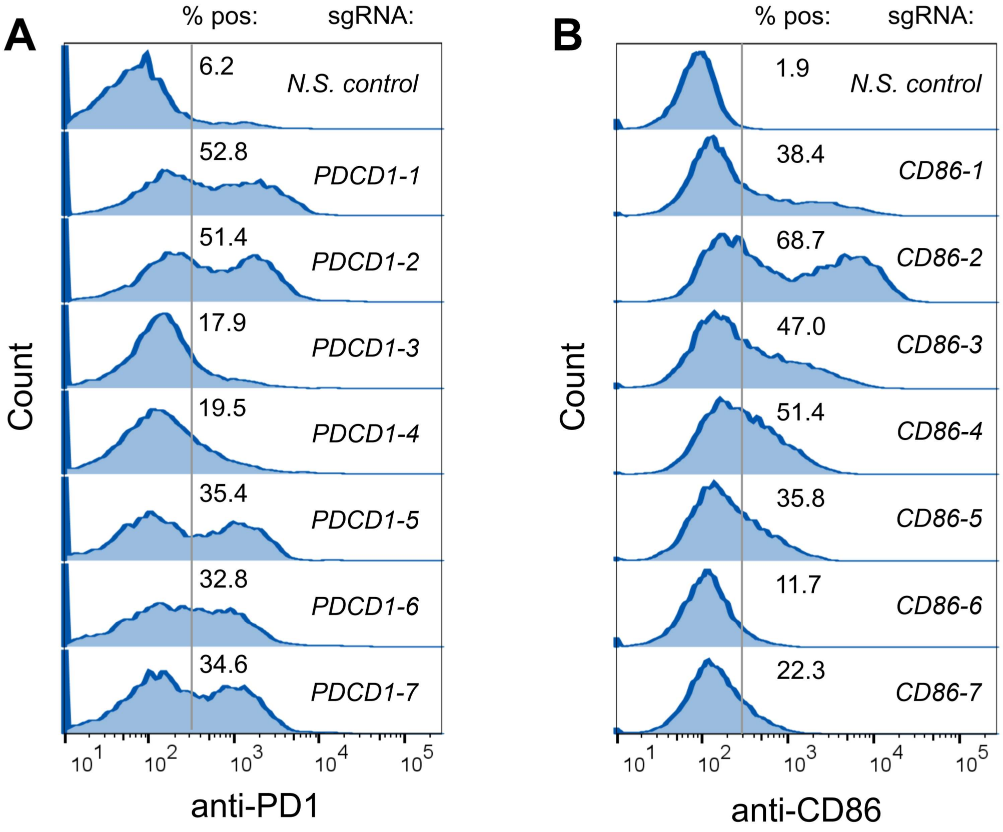

Fig. S2. Analysis of guide activity. A,B. Flow cytometry (FC) was used to monitor surface expression of PD1 (**A**) and CD86 (**B**) 48h following transduction with individual guide RNAs. Representative of three experiments. N.S. : non-specific guide RNA.

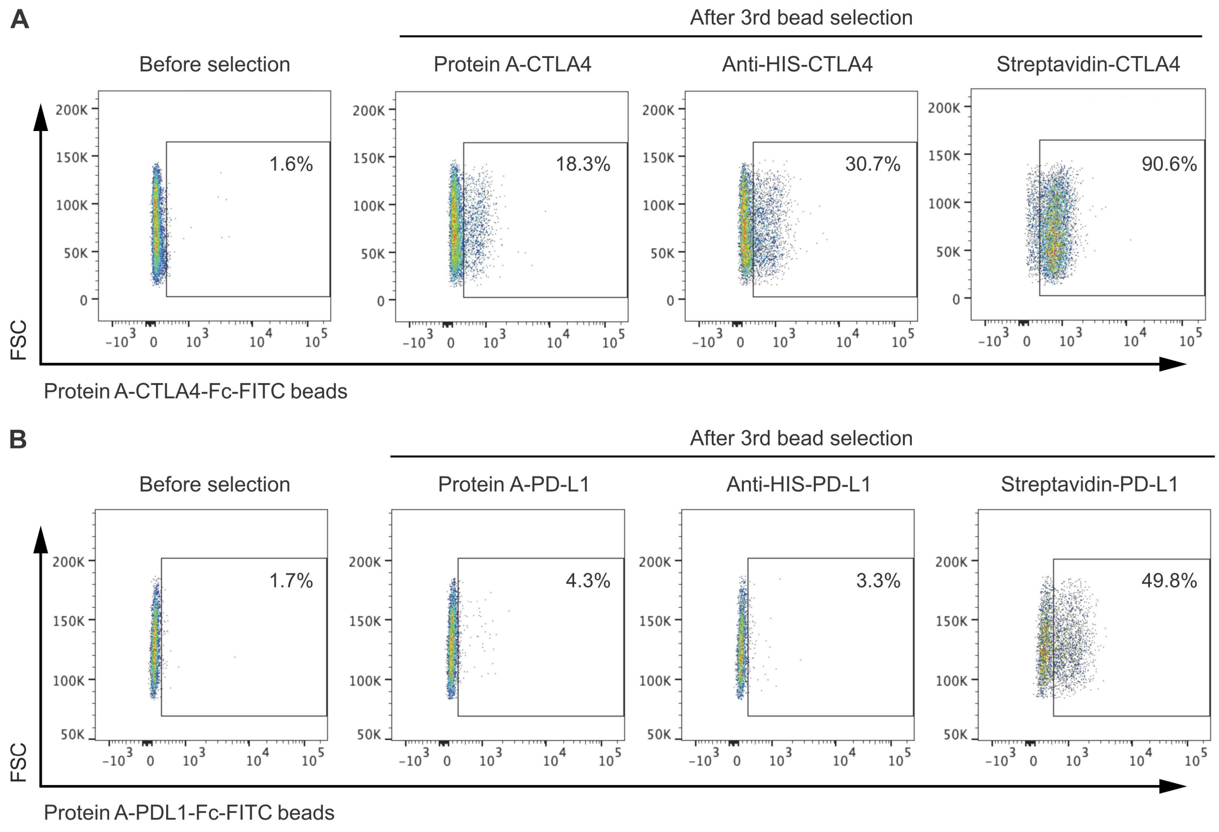

Fig. S3. Serial enrichment monitoring using fluorescently labeled bead bait. A,B. Flow cytometry (FC) was used to monitor binding of FITC-labeled CTLA4- (**A**) or PD-L1- (**B**) bead-bound bait to cells following the 3rd round of serial enrichment.

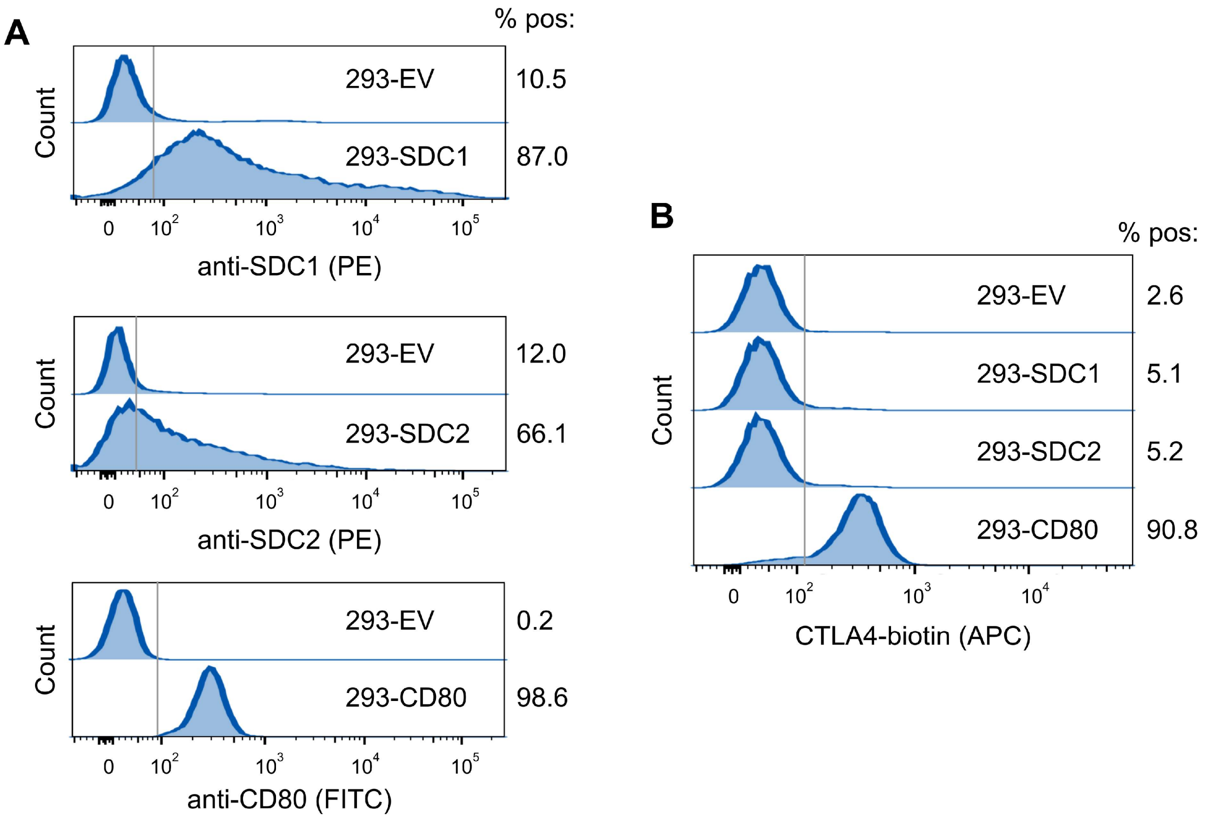

Fig. S4. Evaluation of CTLA4 ectodomain binding to CD80 and syndecans. A. FC was used to verify target expression in 293 cells transfected empty vector (EV) (negative control), expression vectors encoding SDC1 or SDC2 cDNAs, or CD80 activating gRNAs. Data were representative of three experiments. **B.** Soluble recombinant CTLA4-biotin was used to label each of the transfected 293 cells shown in (**A**) and detected using APC-streptavidin. All data were representative of three experiments.

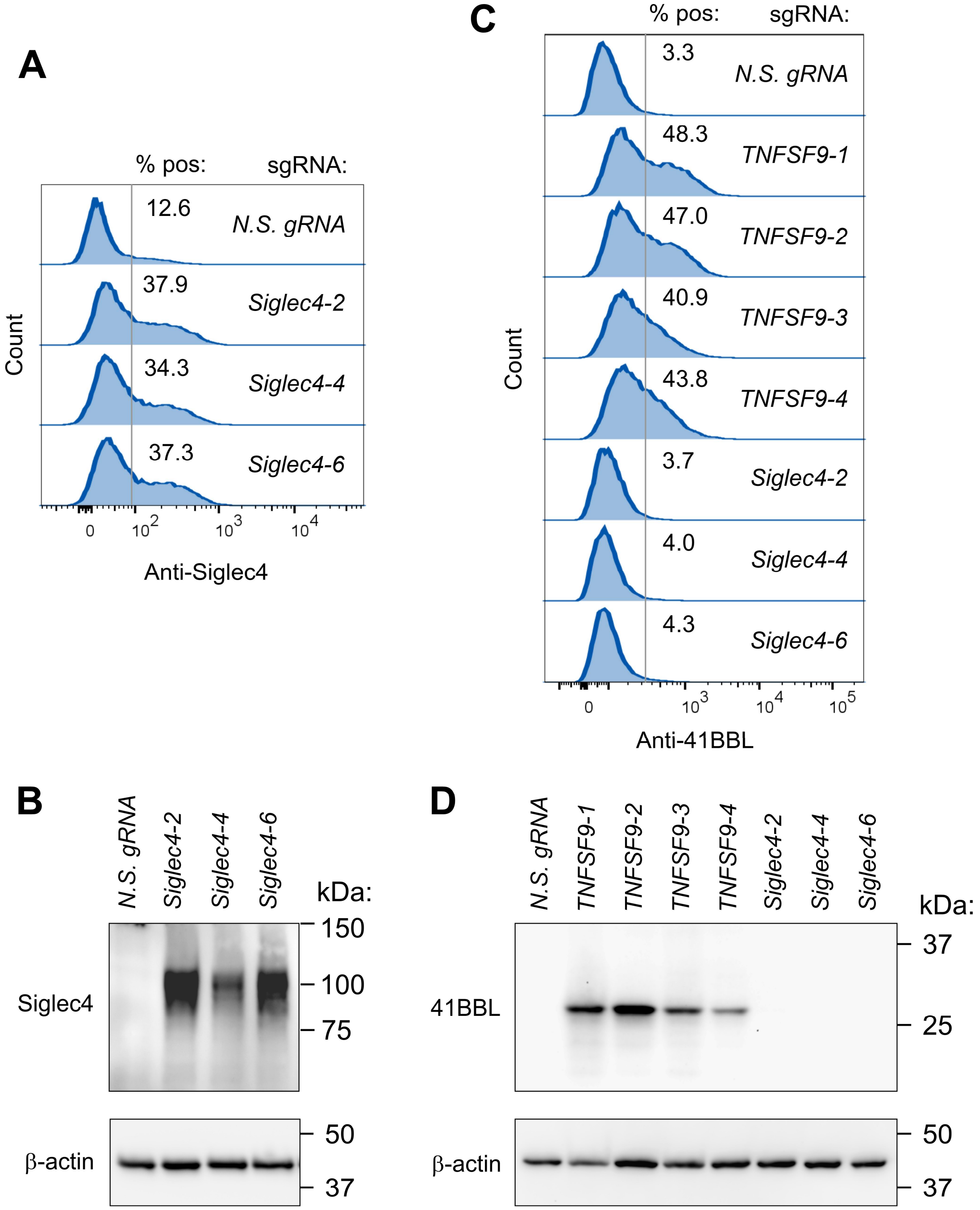

Fig. S5. Analysis of Siglec-4 and 4-1BBL guide activity. Flow cytometry (A,C) and Western blotting (B,D) was used to monitor expression of siglec-4 (A,B) and 4-1BBL (C,D) following transduction with individual guide RNAs. β-actin was used as a loading control. Note that the siglec-4 guides that enriched following 4-1BB selection are unable to activate 4-1BBL expression. All data were representative of three experiments.

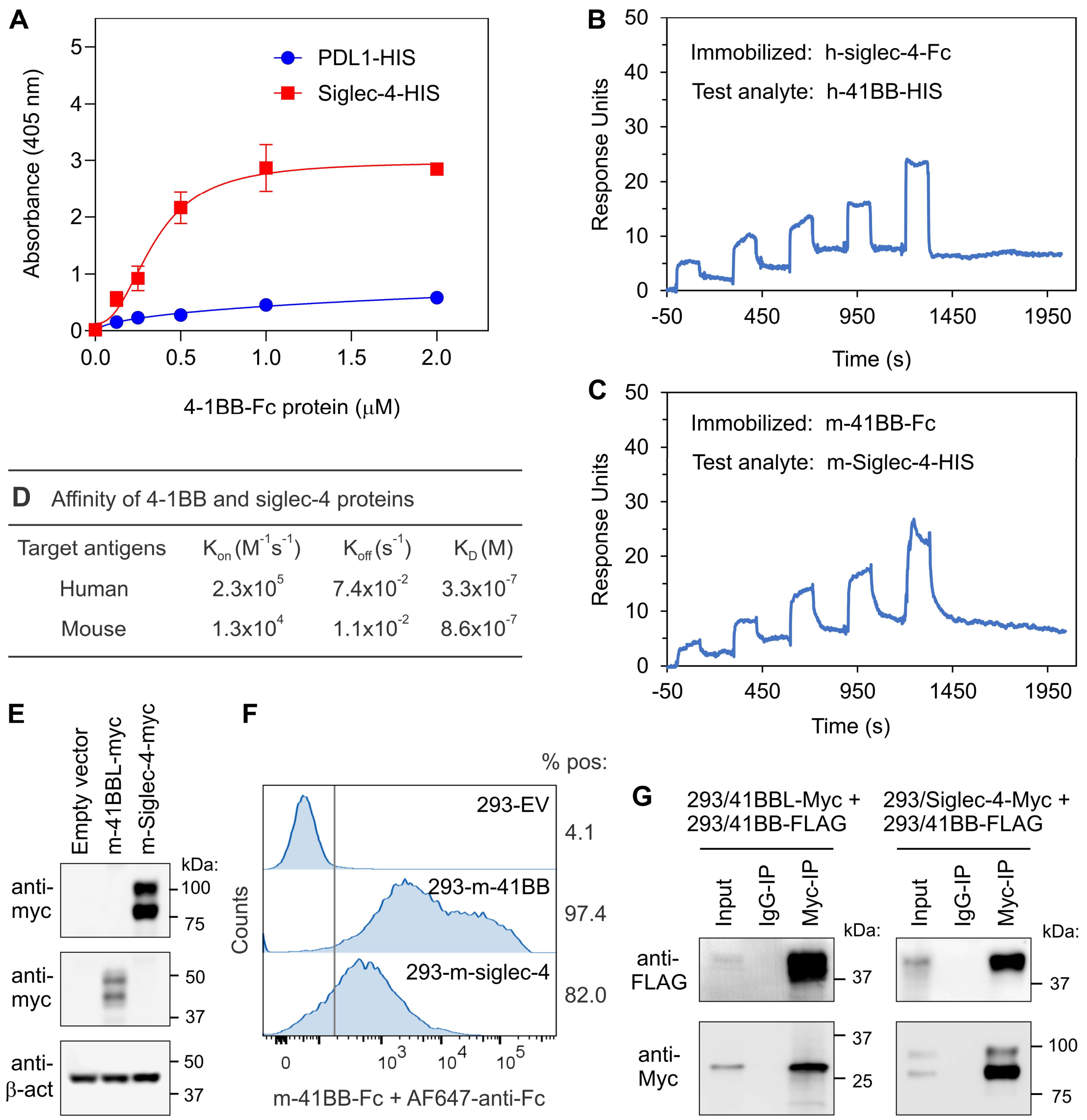

D Affinity of 4-1BB and siglec-4 proteins

Target antigens	K_{on} ($M^{-1}s^{-1}$)	K_{off} (s^{-1})	K_D (M)
Human	$2.3x10^{5}$	$7.4x10^{-2}$	$3.3x10^{-7}$
Mouse	$1.3x10^{4}$	$1.1x10^{-2}$	$8.6x10^{-7}$

Fig. S6. Siglec-4 binds mouse and human 4-1BB. A. ELISA was used to measure binding of recombinant human 4-1BB-Fc protein to immobilized human siglec-4-HIS protein. The results are representative of three different experiments. Data were representative of three experiments. **B,C.** Surface Plasmon Resonance analysis was used to monitor binding kinetics of the recombinant human proteins h-siglec-4 and h-41BB-HIS (**B**) or the mouse proteins m-41BB-Fc and m-siglec-4-HIS (**C**). Data were representative of three experiments. **D.** Table summarizing the on- and off-rates and binding affinity (K_D) values for the human and mouse pairs used in (**B**) and (**C**). **E.** Western blotting was used to verify expression of myc-tagged m-41BBL and m-siglec-4 in 293 cells. Images were representative of three experiments. **F.** FC was used to assess binding of m-41BB-Fc protein to 293 cells expressing empty vector (EV), m-41BB or m-siglec-4. Data were representative of three experiments. **G.** Immunoprecipitation was used to evaluate the interaction between siglec-4 and 4-1BB in cells separately transfected prior to mixing together. Co-cultures of 293-4-1BBL and 293-4-1BB were included as a positive control. Images were representative of three experiments.

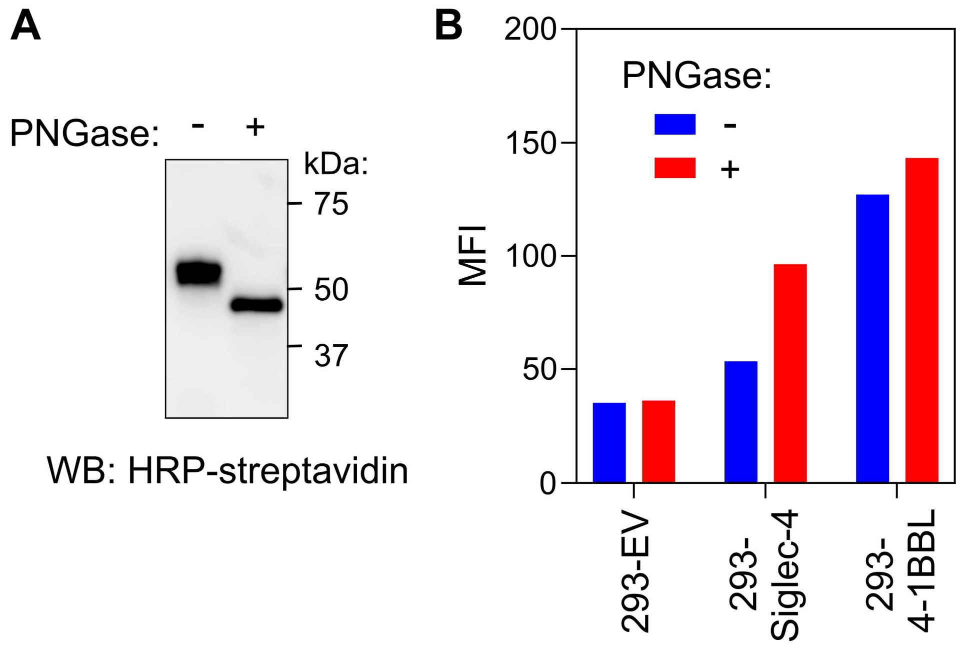

Fig. S7. Impact of 4-1BB deglycosylation on siglec-4 binding. A. Immunoblotting with HRP-streptavidin antibodies was used to assess changes in molecular weight of purified recombinant biotinylated 4-1BB-HIS following treatment with PNGase. Images were representative of three experiments. **B.** FC was used to analyze binding of untreated (- PNGase) or deglycosylated (+ PNGase) biotinylated 4-1BB-HIS protein to 293 cells transfected with EV, siglec-4 or 4-1BBL. Biotinylated 4-1BB-HIS proteins were detected using APC-streptavidin antibodies. MFI: mean fluorescence intensity. Data were representative of three experiments.

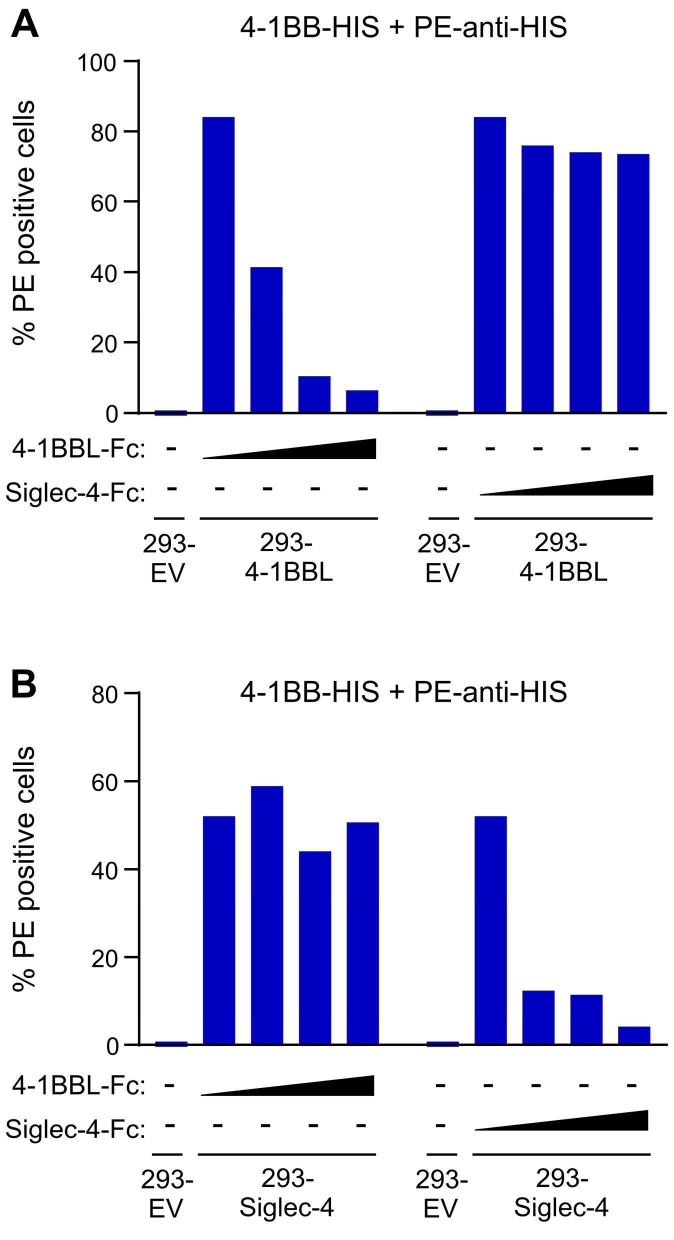

Fig. S8. 4-1BBL and siglec-4 competition assays. A,**B**. FC was used to measure the binding of recombinant 4-1BB-HIS to 293-4-1BBL (**A**) or 293-siglec-4 (**B**) cells in the presence of increasing amount of competitor 4-1BBL-Fc or siglec-4-Fc protein. The wedge indicates an increasing concentration of competitor protein: 0, 0.01, 0.1 or 1 μM. Data were representative of three experiments.

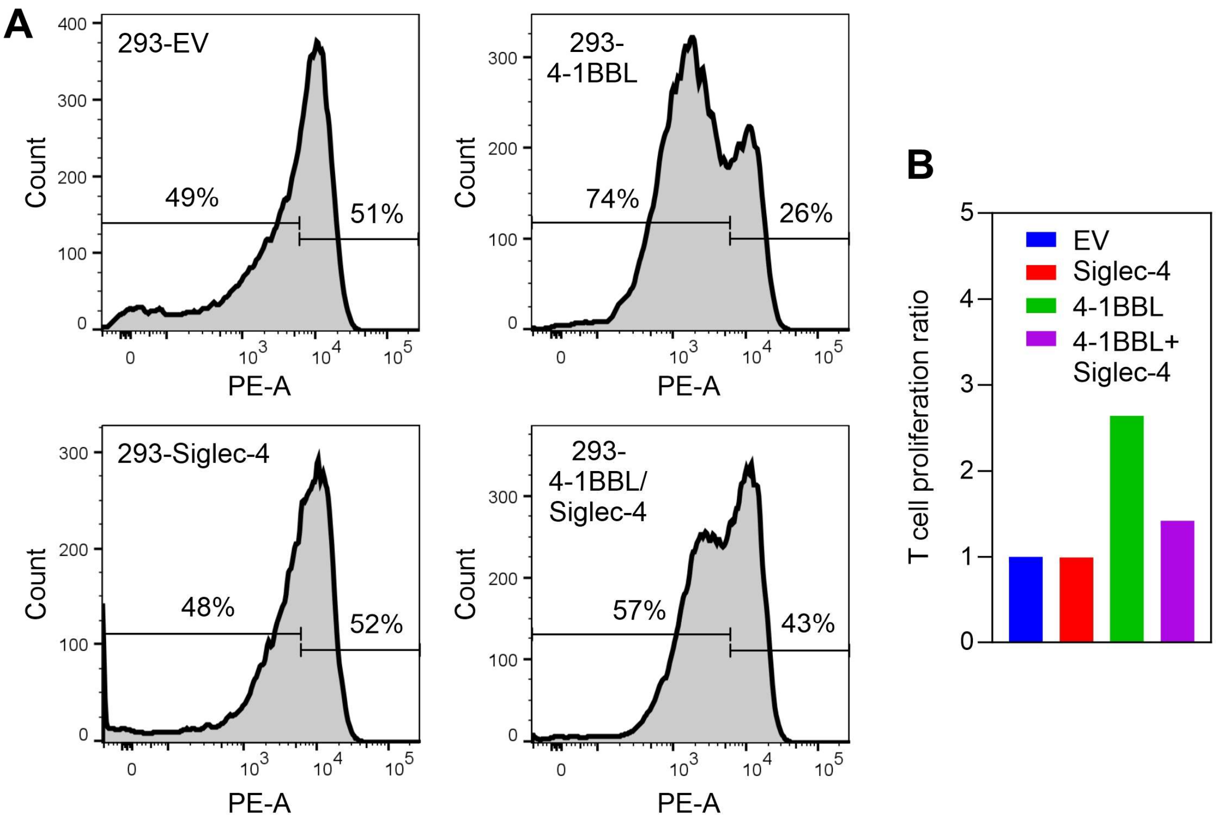

Fig. S9. Proliferation of T cells in 293 co-cultures. A. T cell proliferation was measured by labeling T cells with the vital dye PKH26, mixing 2:5 with various 293 cells, then monitoring the dilution of the cell dye (an indicator of proliferation) by FC 96h later. T cells were co-cultured with 293 cells transfected with empty vector (EV), 4-1BBL, siglec-4, or 4-1BBL plus siglec-4. **B.** Bar graph summarizing the ratio of high proliferating cells to low proliferating cells shown in (**A**). Data were representative of three experiments.

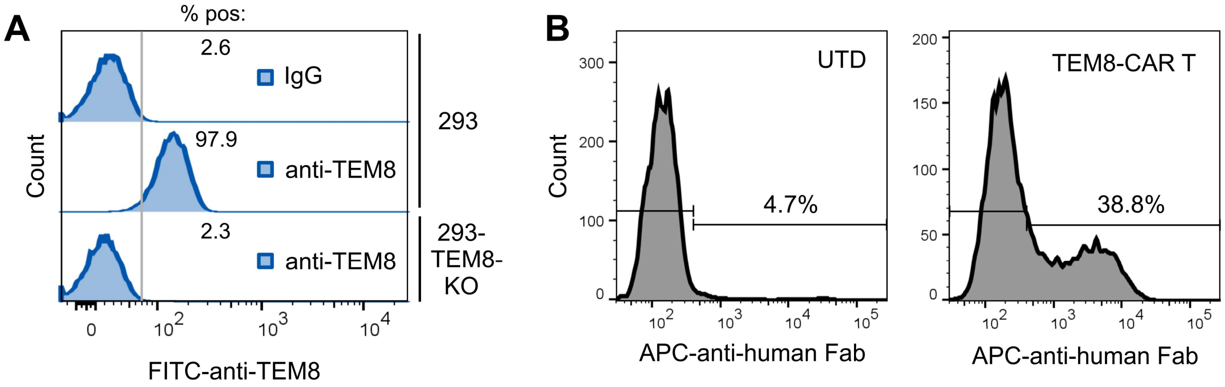

Fig. S10. Generation of TEM8 knockout-target cells and TEM8 CAR T cells. A. FC was used to verify loss of TEM8 expression in 293 cells following CRISPR/Cas9 mediated TEM8 gene inactivation. Data were representative of three experiments. **B.** FC was used to evaluate TEM8 CAR transduction efficiency in primary human T cells. UTD: untransduced T cells. Data were representative of three experiments.

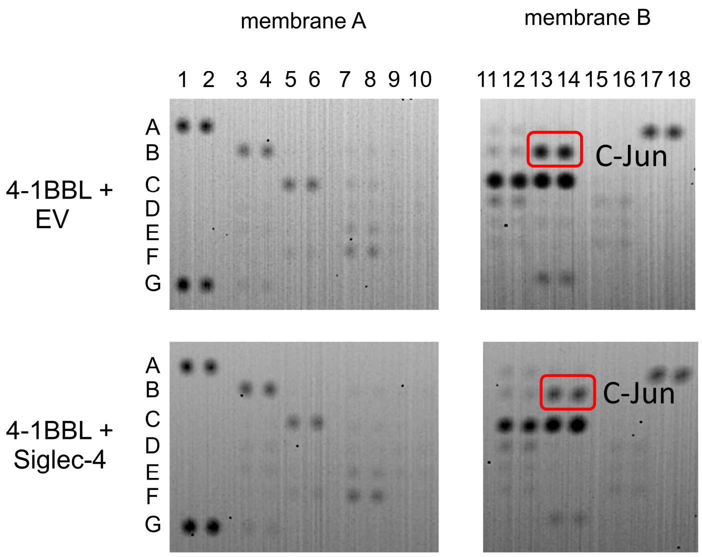

Membrane/ Coordinate	Target/Control	Phosphorylation Site
A-A1, A2	Reference Spot	—
B-A11, A12	Akt 1/2/3	T308
B-A13, A14	Akt 1/2/3	S473
B-A17, A18	Reference Spot	—
A-B3, B4	CREB	S133
A-B5, B6	EGF R	Y1086
A-B7, B8	eNOS	S1177
A-B9, B10	ERK1/2	T202/Y204, T185/Y187
B-B11, B12	Chk-2	T68
B-B13, B14	c-Jun	S63 A
A-C3, C4	Fgr	Y412
A-C5, C6	GSK-3a/ß	S21/S9
A-C7, C8	GSK-3ß	S9
A-C9, C10	HSP27	S78/S82
B-C11, C12	p53	S15
B-C13, C14	p53	S46
B-C15, C16	p53	S392
A-D3, D4	JNK 1/2/3	T183/Y185, T221/Y223
A-D5, D6	Lck	Y394
A-D7, D8	Lyn	Y397
A-D9, D10	MSK1/2	S376/S360
B-D11, D12	p70 S6 Kinase	T389
B-D13, D14	p70 S6 Kinase	T421/S424
B-D15, D16	PRAS40	T246
A-E3, E4	p38α	T180/Y182
A-E5, E6	PDGFRß	Y751
A-E7, E8	PLC-γ1	Y783
A-E9, E10	Src	Y419
B-E11, E12	PYK2	Y402
B-E13, E14	RSK1/2	S221/S227
B-E15, E16	RSK1/2/3	S380/S386/S377
A-F3, F4	STAT2	Y689
A-F5, F6	STAT5a/b	Y694/Y699
A-F7, F8	WNK1	T60
A-F9, F10	Yes	Y426
B-F11, F12	STAT1	Y701
B-F13, F14	STAT3	Y705
B-F15, F16	STAT3	S727
A-G1, G2	Reference Spot	—
A-G3, G4	ß-Catenin	—
A-G9, G10	PBS (Negative Control)	—
B-G11, G12	STAT6	Y641
B-G13, G14	HSP60	—
B-G17, G18	PBS (Negative Control)	—

Fig. S11. A phospho-kinase array was used to assess changes in cell signaling in siglec-4 overexpressing cells. Phospho-kinase arrays were used to assess phosphorylation changes in 293-4-1BB cells co-cultured with 293-4-1BBL cells transiently transfected with either EV (top panel) or siglec-4 (bottom panel). Each phosphoprotein is represented in duplicate. The decrease in c-Jun phosphorylation in response to siglec-4 is highlighted (red box). The phospho-kinase array coordinates for the blots are shown on the table. Data were representative of two experiments.

Supplementary Tables:

Supplementary Tables are provided in separate Microsoft Excel files.

Table S1. Diversity of guide library.
gRNA library following amplification in E. coli and transduction into 293-VM-14.7 cells.

Table S2. Guide library GINI index.
GINI values of gRNA library following amplification in E. coli and transduction into 293-VM-14.7 cells.

Table S3. Stringent guide library.
Using a custom python script and cross-referencing with GuideScan off-target monitoring, up to 5 highly specific gRNAs per gene were identified and combined from the Wright, Calabrese and Weissman libraries to create a stringent library (RC-L-0005). In order not to lose genes due to the absence of highly specific gRNA sequences in their promotors, non-represented genes were identified and guides for them were identified using a more relaxed guidescan threshold (RC-L-0005-Plus). By curating genes from soluble and surface proteome projects, several additional secreted genes absent from the original Wright library were identified and used to create an add-on library (Soluble factor add on).

Table S4. Primer sequences used in the study.
Oligonucleotides used for ligation and primers used for 1st, 2nd and oligo pool PCR.

Table S5. Gene summary from PD-L1 failed dataset.
Gene names, number of different gRNAs per gene, p-values, scores and other values from the Mageck output. This library was selected using Alexa Fluor 488 (AF488)-Strep-PD-L1 (AF488-SA-PDL1) without serial enrichment.

Table S6. gRNA summary from PD-L1 failed dataset.
sgRNA names, gene names, absolute counts in cells with or without selection by AF488-Strep-PDL1, and other values from the Mageck output. This library was selected using AF488-Strep-PDL1 (AF488-SA-PDL1) without serial enrichment.

Table S7. Gene summary from CTLA4 bead based enrichment.
Gene names, number of different gRNAs per gene, p-values, scores and other values from the Mageck output. These library values were derived following the 1st, 2nd or 3rd enrichment using biotinylated CTLA4 bound to streptavidin beads (SA-CTLA4) or following the third enrichment using HIS-tagged CTLA4 bound to anti-HIS beads (αHis-CLTA4).

Table S8. gRNA summary from CTLA4 bead based enrichment.
sgRNA names, gene names, absolute counts in cells with or without selection, and other values from the Mageck output. These library values were derived following the 1st, 2nd or 3rd enrichment using biotinylated CTLA4 bound to streptavidin beads (SA-CTLA4) or following the third enrichment using HIS-tagged CTLA4 bound to anti-HIS beads (αHis-CLTA4).

Table S9. gRNA summary from PD-L1 bead based enrichment.
sgRNA names, gene names, absolute counts in cells with or without selection, and other values from the Mageck output. These library values were derived following the 1st, 2nd or 3rd enrichment using biotinylated PD-L1 bound to streptavidin beads (SA-PDL1) or following the third enrichment using HIS-tagged PD-L1 bound to anti-HIS beads (αHis-PDL1).

Table S10. Gene summary from PD-L1 bead based enrichment.
Gene names, number of different gRNAs per gene, p-values, scores and other values from the Mageck output. These library values were derived following the 1st, 2nd or 3rd enrichment using biotinylated PD-L1 bound to streptavidin beads (SA-PDL1) or following the third enrichment using HIS-tagged PD-L1 bound to anti-HIS beads (αHis-PDL1).

Table S11. Gene summary from PD-L1 bead based enrichment using new stringent library.
Gene names, number of different gRNAs per gene, p-values, scores and other values from the Mageck output. These library values were derived following the 1st, 2nd or 3rd enrichment using biotinylated PD-L1 bound to streptavidin beads (SA-PDL1).

Table S12. gRNA summary from PD-L1 bead based enrichment using new stringent library.
sgRNA names, gene names, absolute counts in cells with or without selection, and other values from the Mageck output. These library values were derived following the 1st, 2nd or 3rd enrichment using biotinylated PD-L1 bound to streptavidin beads (SA-PDL1).

Table S13. Gene summary from 4-1BB bead based enrichment.
Gene names, number of different gRNAs per gene, p-values, scores and other values from the Mageck output. These library values were derived following the 1st, 2nd, 3rd or 4th enrichment using biotinylated 4-1BB bound to streptavidin beads (SA-4-1BB).

Table S14. gRNA summary from 4-1BB bead based enrichment.
sgRNA names, gene names, absolute counts in cells with or without selection, and other values from the Mageck output. These library values were derived following the 1st, 2nd, 3rd or 4th enrichment using biotinylated 4-1BB bound to streptavidin beads (SA-4-1BB).
